# Eggshell and environmental bacteria contribute to the intestinal microbiota of growing chickens

**DOI:** 10.1186/s40104-020-00459-w

**Published:** 2020-06-11

**Authors:** Joel J. Maki, Elizabeth A. Bobeck, Matthew J. Sylte, Torey Looft

**Affiliations:** 1 Food Safety and Enteric Pathogens Research Unit, National Animal Disease Center, Agricultural Research Service, US Department of Agriculture, 1920 Dayton Ave, Ames, IA 50010 USA; 2 Interdepartmental Microbiology Graduate Program, Iowa State University|, Ames, IA 50011 USA; 3 Oak Ridge Institute for Science and Education, ARS Research Participation Program, Oak Ridge, TN 37830 USA; 4 Department of Animal Science, Iowa State University, Ames, IA 50011 USA

**Keywords:** 16S rRNA gene, Eggshell microbiota, Environmental microbiota, Hatching, Intestinal colonization, SCFA, Succession

## Abstract

**Background:**

The initial intestinal microbiota acquired from different sources has profound impacts on animal health and productivity. In modern poultry production practices, the source(s) of the establishing microbes and their overall contribution during development of gastrointestinal tract communities are still unclear. Using fertilized eggs from two independent sources, we assessed the impact of eggshell- and environmental-associated microbial communities on the successional processes and bacterial community structure throughout the intestinal tract of chickens for up to 6 weeks post-hatch.

**Results:**

Culturing and sequencing techniques identified a viable, highly diverse population of anaerobic bacteria on the eggshell. The jejunal, ileal, and cecal microbial communities for the egg-only, environment-only, and conventionally raised birds generally displayed similar successional patterns characterized by increasing community richness and evenness over time, with strains of *Enterococcus, Romboutsia*, and unclassified Lachnospiraceae abundant for all three input groups in both trials. Bacterial community structures differed significantly based on trial and microbiota input with the exception of the egg-exposed and conventional birds in the jejunum at week 1 and the ileum at week 6. Cecal community structures were different based on trial and microbiota input source, and cecal short-chain fatty acid profiles at week 6 highlighted functional differences as well.

**Conclusion:**

We identified distinct intestinal microbial communities and differing cecal short-chain fatty acid profiles between birds exposed to the microbiota associated with either the eggshell or environment, and those of conventionally hatched birds. Our data suggest the eggshell plays an appreciable role in the development of the chicken intestinal microbiota, especially in the jejunum and ileum where the community structure of the eggshell-only birds was similar to the structure of conventionally hatched birds. Our data identify a complex interplay between the eggshell and environmental microbiota during establishment and succession within the chicken gut. Further studies should explore the ability of eggshell- and environment-derived microbes to shape the dynamics of succession and how these communities can be targeted through interventions to promote gut health and mitigate food-borne pathogen colonization in poultry.

## Introduction

Within the gastrointestinal tract (GIT) of chickens, there is a complex and dynamic interaction between the host and the rich microbial community present. The interplay of these two components is responsible for the breakdown of foodstuffs, proper nutrient absorption, growth, and health [1]. Microbial succession within the poultry GIT can impact microbiota structure, as well as host nutrient absorption and physiological processes, and early colonization events are important drivers of host health [2–4]. The source and composition of microbes introduced to newly hatched chicks likely affects the development of intestinal microbiota.

Intestinal microbiota members can be acquired vertically, horizontally, and from the environment [5]. In mammals, vaginal birth is a major route for transmission of commensal microorganisms to offspring [6, 7]. Additional postnatal microbial acquisition may occur from diet, interaction with the mother, and the immediate environment, resulting in microbiota establishment and succession within the GIT [8]. Once bacteria are introduced into the GIT, successional processes are fairly consistent across livestock species. Pioneering facultative anaerobes, such as *Escherichia* spp. and *Enterococcus* spp., help create an environment that permits the establishment of strict anaerobic bacteria such as *Clostridium* spp. and other members of the phylum Firmicutes [9–12].

The source of microbiota colonizing poultry GIT has likely changed as production practices modernized. Prior to commercialization of poultry production, hens maintained contact with eggs within the nest throughout the incubation and hatching periods. The continued contact provided newly hatched chicks with a continuous source of maternal microbiota from the hen itself and the nest environment, which is also rich in maternally derived microbiota [13]. In modern poultry production practices, eggs are hatched without hen contact, limiting the opportunity for vertical transmission of microbiota to the chicks [14]. This separation potentially impacts microbial succession processes within the GIT, calling into question where chicks acquire their intestinal microbiota. Several studies have determined the successional processes at play early in the poultry GIT establishment process; many with the assumption the gut microbiota is primarily acquired from the environment, diet, and animal handlers post-hatch due to the common practices of washing and fumigating incubators, as well as the chemical disinfection of the eggs at commercial chicken hatcheries [2, 10, 13, 15–19]. To our knowledge, only one study has assessed the potential impact of vertical transmission as a source of commensal microbiota [20], though *in ovo* transfer of *Mycoplasma*, *Salmonella*, and other potential pathogens has been previously observed [21–23].

The eggshell is a potential source of microbial inoculum for hatching chicks. Prior to oviposition, eggs are coated in a diverse consortium of microbes as they pass through the hen reproductive tract as well as the distal digestive tract (cloaca) [24]. Microbial succession within complex biofilms suggests that founder microbial species play an important role by modifying the local environment in a way that mediates filtering of further microbial inputs later in the successional process [25]. The maternal microbiota deposited on the eggshell could be a potential source of important founder species, setting the stage for a healthy GIT microbiota. In order for eggshell microbes to colonize hatching chicks, bacteria would need to survive on the highly oxygenated and nutrient-poor eggshell and cuticle through the incubation period (21 days in chickens), or penetrate the eggshell and eggshell membranes. A majority of the anaerobic commensals present in human fecal samples are able to sporulate [26], and the same is likely true for poultry commensals. Thus, obligate anaerobes originating from the poultry intestinal tract could survive the harsh conditions on the eggshell surface in a sporulated form to be ingested by a hatching chick at hatch.

Whether microbes from the eggshell or the environment serve as the dominant driver for microbiota colonization and succession in poultry remains to be determined. Our lab previously developed an approach to sterilize and hatch eggs in germ-free isolators [27], a system that could be adapted to interrogate intestinal microbial succession in poultry. Non-sterilized eggs hatched in a sterile isolator (containing sterilized water, feed, and litter) would prevent environmental bacteria (other than what is present on the eggshell) from being introduced into the chicken GIT. Conversely, sterilizing the surface of the eggshell eliminates the bacteria deposited on the eggshell by the hen, allowing succession to occur with only environmental microbes.

In this study, we sought to assess the eggshell and environment as sources of microbial communities contributing to successional processes in the chicken intestinal tract for up to 6 weeks post-hatch. We followed microbial succession within the small intestine (jejunum and ileum), large intestine (ceca), and feces of birds hatched from eggs originating from two different flocks. Eggs were separated into microbiota input groups that were exposed to microbes from only the eggshell, only the environment, or both. The overall goal was to identify the contribution of the eggshell versus environment as microbiota sources for newly hatched chicks, and understand how the two microbiota sources impacted the development and functionality of the GIT microbial communities as birds matured. Additionally, we sought to determine the composition and confirm viability of bacteria on the chicken eggshell. Both the eggshell and the environment provided growing chicks with a source of viable bacteria, producing GIT communities with unique compositions and functional capabilities, suggesting both the eggshell and the environment contribute to the establishment of and succession within the chicken GIT microbiota.

## Methods

### Animal experimental design

Protocols for this study were approved and conducted according to National Animal Disease Center (NADC, Ames, IA, USA) Institutional Animal Care and Use Committee protocol. Chicken eggs were obtained from two different sources, and hatched under the same conditions for two independent experiments. Animal numbers vary between microbiota input groups and trials (15–51 birds/group, average = 34) due to variable egg fertilization and hatch rates (see Table S1). The eggs for the first trial (T1) were obtained from a research flock of crossbred white leghorn layers at Iowa State University (ISU). To limit environmental contamination, eggs were collected shortly after oviposition using sterile surgeon’s gloves, and placed in sterile Duraporter Transport Boxes (Heathrow Scientific, Vernon Hills, IL, USA). After transport to the NADC, eggs were randomly assigned into one of three microbiota input groups. The eggs (white leghorn) for the second trial (T2) were obtained from a commercial hatchery (IA, USA), and transported to the NADC. Upon arrival, eggs were randomly assigned into the three microbiota input groups using sterile surgeon’s gloves. Post-hatch birds in all groups were fed irradiated starter feed, formulated for chicks (Harlan Laboratories, Madison, WI, USA) and provided water ad libitum.

**Fig. 1:**
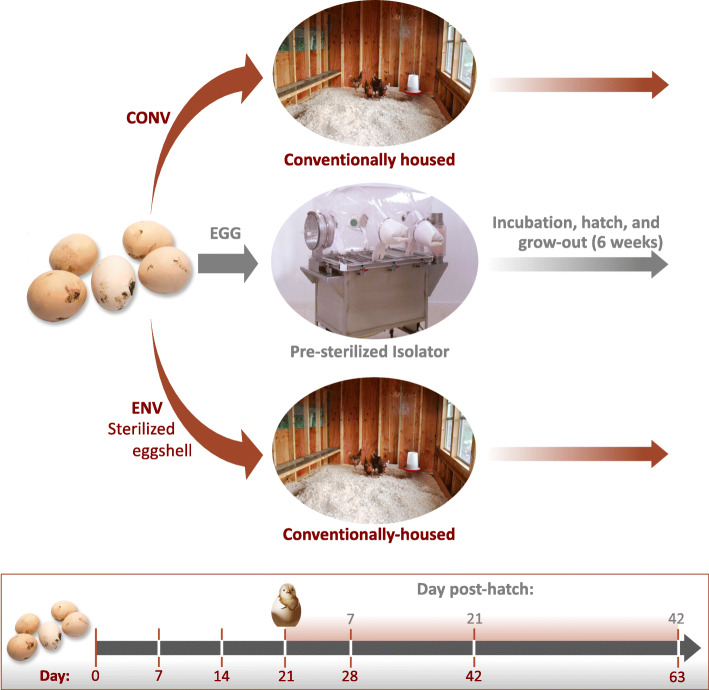
Experimental design for both trials. Total numbers of days since the eggs were received are indicated in red on the timeline. The numbers of days post-hatch are indicated in grey on the timeline

In the conventional input group (CONV), birds were exposed to both the eggshell microbiota and the environmental microbiota (Fig. 1). Eggs in CONV input group were incubated for 21 days in Ovation 56 EX fully automatic digital egg incubators (Brinsea, Titusville, FL, USA) at 37.6 °C and 55–70% humidity. Virkon-S (DuPont Animal Health Solutions, Wilmington, DE, USA) was used to disinfect incubators before use. Upon hatch, chicks were removed from the incubator and placed on fresh litter. A cohort of birds was leg banded and used for repeated collection of weekly fecal swabs (6 weeks total). Non-banded birds were randomly selected at weeks 1 and 3, and the banded birds selected at week 6, and euthanized by CO_2_ gas asphyxiation, followed by necropsy for collection of intestinal tissues and contents. Birds in the eggshell-only microbiota input group (EGG) had the eggshell microbiota as the sole inoculum source for hatching chicks and were deprived of environmental microbiota (Fig. 1). Eggs in the EGG group were aseptically placed in sterilized Duraporter Transport Boxes before passage into a sterile, vinyl germ-free isolator. The isolator was previously loaded with two egg incubators, double autoclaved dH_2_O jugs, irradiated feed, sterile litter, and miscellaneous supplies. The isolator and contents were sterilized with chlorine dioxide gas (700 ppm terminal concentration). Duraporters containing eggs were sealed, placed in the isolator entry port, and surface sterilized with chlorine dioxide for 10 min prior to introduction into the germ-free isolator. Eggs were incubated for 21 days as described above. Upon hatch, chicks were removed from the incubator and placed on sterilized litter in the isolator basin. Birds were provided feed and water ad libitum. A cohort of birds in the isolator was leg banded and used for the collection of weekly fecal swabs. Non-cohort birds were randomly selected for euthanasia and necropsy for sampling at weeks 1 and 3, and the banded birds selected at week 6, as described above. Birds in the environment-only microbiota input group (ENV) were deprived of the eggshell microbiota but allowed to acquire microbes from the environment (Fig. 1). Eggs in the ENV group were surface sterilized, as previously described, omitting the terminal immersion in betadine [27]. Eggs were sequentially immersed in warm (32.8 °C) acidified bleach (pH 5.4; Clorox, University Park, IL, USA) and diluted (1:18:1; base:dH_2_O:activator) chlorine dioxide (Clidox-S, Pharmacal Research Labs, Naugatuk, CT, USA) for 10 min each with a 5-min incubation at 22.0 °C between the two immersions. Culturing of whole eggs confirmed successful sterilization. Post-sterilization, eggs were placed in an Ova-Easy 380 Advance Series II Cabinet Incubator (Brinsea, Titusville, FL, USA), and incubated using the same parameters described above. Upon hatch, chicks were removed from the incubator and placed on fresh, non-sterilized litter. Birds were provided feed and water ad libitum*.* Cohorts of birds were leg banded and used for the collection of weekly fecal swabs and randomly selected, non-leg banded birds were necropsied for sample collection at the time points described above.

At necropsy, cecal, ileal, and jejunal lumenal contents and mucosal swabs (Foam-over-cotton; VWR, Radnor, PA, USA) were collected from each bird for microbiota analysis. Analysis of short-chain fatty acid (SCFA) composition was performed on 1 g of cecal contents from leg banded birds, at the terminal necropsy of T2 (Table S1). All samples were kept on ice prior to processing. Cecal contents for SCFA analysis were immediately frozen upon return to the lab. Intestinal samples for microbiota analysis were distributed into 96 deep well plates and frozen prior to DNA extraction. Fecal swabs (Knitted polyester-tipped; Puritan, Guilford, ME) were placed into sterile 1.5 mL microcentrifuge tubes with 200 μL of sterile phosphate-buffered saline (1x, PBS) (Sigma, Darmstadt, Germany), vortexed, and frozen prior to DNA extraction.

### DNA isolation and cultivation of viable anaerobically growing bacteria from the chicken eggshell

DNA was isolated from egg swabs of the T1 eggs. Knitted polyester-tipped swabs were submerged in sterile 1× PBS and vortexed to suspend cells prior to DNA extraction. A different quadrant of each egg was swabbed at each subsequent sampling point. Sample numbers and times are listed in Supplemental Table S1.

Eggs from T2 were collected for both DNA isolation and anaerobic culturing. Eggs were aseptically removed from the CONV room incubators at each timepoint and placed in sterile Whirl-Pak bags (Nasco, Fort Atkinson, WI, USA) before 20 mL of sterile 1× PBS was added and the egg was gently massaged to suspend bacterial cells from the eggshell surface. Sampling times and numbers are described in Supplemental Table S1. The PBS supernatant was then collected and added to a new, sterile 50 mL conical tube (Falcon, Fisher Scientific, Hampton, NH, USA) and centrifuged to pellet bacterial cells. Pellets were resuspended in 1 mL of sterile PBS, of which 500 μL was added to a sterile 1.5 mL microcentrifuge tube and passed into an anaerobic chamber (Coy Lab Products, Grass Lake, MI, USA). Isolation of sporulating, anaerobically growing bacteria was conducted as described previously [26]. Briefly, half of the above aliquot (250 μL) of egg wash bacteria was treated 1:1 with 70% ethanol for 4 h to kill vegetative cells. This suspension was then serially diluted and plated onto Brain Heart Infusion (BHI) agar (VWR, Radnor, PA, USA) supplemented with 0.1% (v/v) whole chicken bile. Total viable, anaerobically growing bacteria were determined via serial-dilution and plating of the untreated egg wash aliquot (250 μL) on BHI + 0.1% whole chicken bile agar plates. All plates were incubated anaerobically at 42 °C. Colonies were counted after 72 h of incubation to determine colony-forming units (CFU)/eggshell. The other 500 μL of the resuspended eggshell bacterial cell pellet was utilized for DNA extraction. Colony forming units/eggshell values were compared between time points using the pairwise.t.test function (Benjamini-Hochberg correction) in the statistical computing software R v3.5.1 [28].

### DNA extraction from eggshells and tissues

Genomic DNA was isolated from eggshell washes, fecal swabs, and intestinal lumen and mucosa samples using the MagAttract PowerMicrobiome 96-well DNA/RNA kit (Qiagen, Hilden, Germany) following the manufacture’s instructions. The V4 hypervariable region of the bacterial 16S rRNA gene was PCR amplified and sequenced using the paired-end method on the MiSeq platform (Illumina, San Diego, CA, USA) as previously described [17, 20].

### 16S rRNA gene analysis

Sequences were imported into R and dada2 v1.11.3 [29] was used to generate a count table of amplicon sequence variants (ASVs). Taxonomy was assigned using the Silva reference database v132 [30] using previously described methods [31]. The Phyloseq package v1.26.1 [32] was used to filter out poor performing samples, subset samples to 1073 reads, assess relative abundance for ASVs, determine alpha diversity measures, and to curate the dataset for statistical tests and visualizations. The Vegan package v2.5–5 [33] was used for pairwise permutational multivariate analysis of variance (PERMANOVA) and permutation multivariate analysis of dispersion (PERMDISP) calculations. Resulting *P*-values were corrected for the false discovery rate. Dissimilarity matrices were constructed comparing samples using Bray-Curtis, which were visualized with Non-Metric Multidimensional Scaling (NMDS) ordinations. The “venn” function in gplots v3.0.1.1 [34] was used to identify ASVs that were conserved between or unique to input groups and egg sources. ASVs required at least 10 reads for a given microbiota input group to be included in this assessment. The core microbiota were identified as ASVs present in all groups at a relative abundance of at least 0.1%. Comparisons for alpha diversity metrics were made using an analysis of variance (ANOVA) followed by subsequent pairwise comparisons with the Tukey “Honest Significant Difference” method with *P*-values adjusted for the false discovery rate. Comparisons between groups were deemed significant if q < 0.05.

### SCFA analysis

Short-chain fatty acid concentrations in 1 g of cecal contents collected from the T2 birds at week 6 were quantified using previously described methods on an Agilent 7890 Gas Chromatograph (Agilent, Santa Clara, CA, USA) [35, 36]. The data were imported into R and the Tukey-based method described above was used to compare the SCFA profiles between the treatments. As above, comparisons between groups were deemed significant if *P* < 0.05.

## Results

### Culturing from eggs

**Fig. 2:**
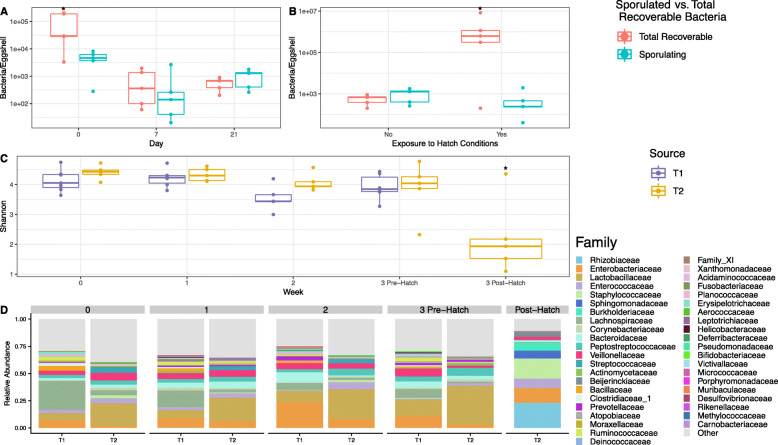
Description of the eggshell-associated microbial communities using culture-based and sequencing-based techniques. **a** Boxplot tracking pre-hatch changes in the abundance (CFU/eggshell) of total recoverable anaerobically growing bacteria (red) and recoverable suspected sporulating anaerobic bacteria (blue) throughout incubation. Whiskers represent the maximum and minimum values. The interquartile range is indicated by the upper and lower boundaries of the boxes. Significance between the total recoverable anaerobic bacteria counts and the suspected sporulating bacteria counts and between weeks was determined by pairwise T-test. An asterisk (*) indicates a significant difference in bacterial counts between both the total viable anaerobe and recoverable sporulating populations within a timepoint and/or between that time point and other time points (*P* < 0.05). **b** Boxplot comparing abundance (CFU/eggshell) of total recoverable anaerobically growing bacteria (red) and recoverable suspected sporulating anaerobic bacteria (blue) at day 21 both pre-hatch and post-hatch. Significance between the total recoverable anaerobic bacteria counts and the suspected sporulating bacteria counts and between pre- and post-hatch was determined by pairwise T-test. An asterisk (*) indicates a significant difference in bacterial counts between both the total viable anaerobe and recoverable sporulating populations within a time point and between that time point and other time points for that bacterial population (*P* < 0.05). **c** Changes in eggshell microbiota alpha-diversity (Shannon index) throughout the incubation period for both trials. Week 3 post-hatch community analysis was not conducted for T1 eggs. Comparisons between Shannon indices between weeks and trials were made using an analysis of variance (ANOVA) followed by subsequent pairwise comparisons with the Tukey’s “Honest Significant Difference” method. An asterisk (*) indicates a significant difference in Shannon index between that time point and other time points within the same trial (*P* < 0.05). **d** Stacked barcharts comparing the weekly relative abundance of bacterial families representing > 0.01 (1%) of the eggshell bacterial community at any time point within either T1 or T2

Eggs washes from trial 2 (T2) assessed the number of total anaerobes and spore-forming anaerobes present on the eggshell surface (Fig. 2a-b). Upon arrival to the NADC (day 0), eggshell samples had high numbers of anaerobic bacteria (91,903 ± 44,067, mean ± standard error of the mean) present on the eggshell surface. A smaller number of those bacteria (4,600 ± 1,320) resisted ethanol treatment, suggesting they were likely spore-forming. Total detected anaerobes dropped by day 7 of egg incubation and remained low while the number of spore-formers was constant (Fig. 2a). On eggshells, after the hatching process completed, the total number of anaerobic bacteria significantly increased to an average of (2,063,240 ±1,555,621) CFU/eggshell (Fig. 2b). However, this increase did not reflect an increase in the number of spore-forming anaerobes, which stayed within the consistent CFU/eggshell range observed throughout the incubation process (584 ± 345).

### 16S rRNA gene microbiota analysis

Sequencing of the V4 region of the 16S rRNA gene yielded a total of 56,712,132 paired–end reads from the 1,544 T1 and T2 samples that survived initial filtration (of a total 1,774 samples for both trials combined). The mean sequence number ± standard error of the mean was 36,731 ± 815. A total of 5,738 unique ASVs were identified in the post-processing samples.

### Eggshell 16S rRNA gene analysis

Alpha diversity, measures of community richness and evenness (Shannon index), was assessed for the microbial communities present on the eggshells. Alpha diversity did not vary significantly by trial or week, with the exception of the week 3 post-hatch eggshell community for the T2 trial, which was significantly less rich than all pre-hatch eggshell communities (Fig. 2c). Firmicutes dominated the T1 eggs at all time points (Figure S1). Lachnospiraceae was the most abundant family on the T1 eggshells at weeks 0 and 1. Enterobacteriaceae became the dominant family at week 2 and Lactobacillaceae was the most abundant at week 3 (pre-hatch) (Fig. 2d). Firmicutes also dominated the T2 eggs, with the exception of the T2 eggshell community post-hatching, which was > 50% Proteobacteria (Figure S1). Lactobacillaceae was highly abundant for all weeks pre-hatch for the T2 eggs (Fig. 2d). Rhizobiaceae became the most abundant bacterial family detected on the eggshell surface after hatching (Fig. 2d). Relative abundance plots at the genus level are displayed in Supplemental Figure S1.

A core eggshell microbiota was determined by identifying ASVs present in at least one of the trials at > 10 reads. Of the ASVs present at > 0.1% abundance, 50 were unique to the T1 eggshells, 59 were unique to the T2 eggshells, and 55 ASVs were shared between the trials, with some of the more highly abundant ASVs belonging to genera such as *Lactobacillus, Enterococcus*, *Romboutsia,* and *Escherichia* (Table S2).

Beta diversity, the variation in community composition, across eggshell communities was compared with PERMANOVA. Trial significantly impacted the community composition on the eggshell, with T1 and T2 eggs being significantly different (q < 0.05) at all weeks, though there were no significant differences in the beta dispersion, the average distance of individual communities to the group centroid, based on PERMDISP (Table S3). Comparing between weeks within a trial, T1 eggshell community composition was only different when comparing the week 0 and week 2 eggshell communities (q < 0.05) (Table S3). The average distance of individual communities to the group centroid between weeks were not significant (*P* > 0.05) (Table S3). The T2 eggshell community compositions at week 3 (both pre- and post-hatching) were significantly different than all other weeks and from one another (q < 0.05) (Table S2). Beta dispersion was significantly different between the week 2 and week 3 post-hatch bacterial communities (*P* < 0.05) (Table S3).

### Fecal swab 16S rRNA gene analysis

**Fig. 3:**
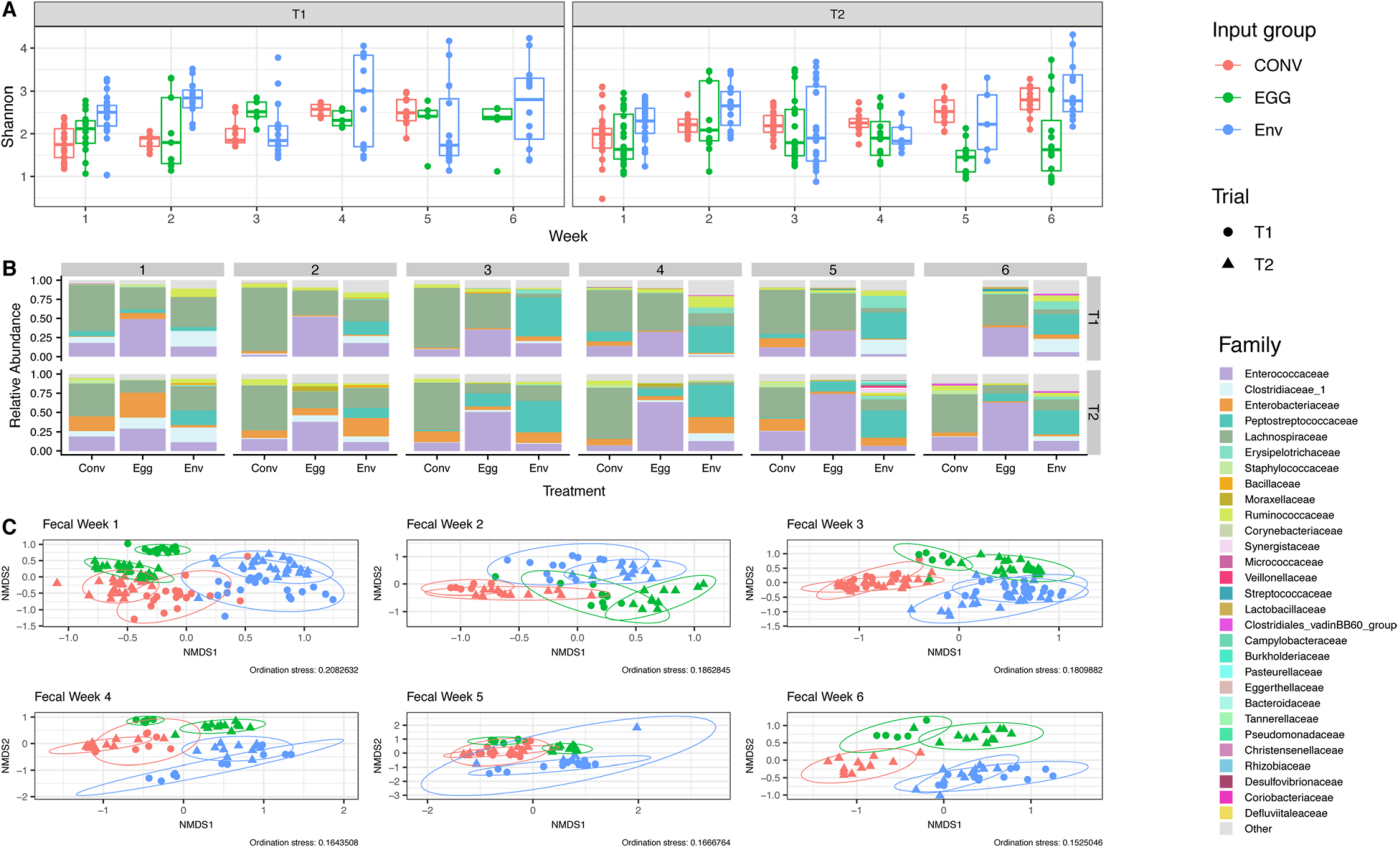
Comparisons of fecal swab bacterial community composition throughout both T1 and T2. **a** Changes in fecal swab microbiota alpha-diversity (Shannon index) for different microbiota input groups throughout the 6-week trial period for both T1 and T2. Comparisons between Shannon indices between microbiota input groups, weeks, and trials were made using an analysis of variance (ANOVA) followed by subsequent pairwise comparisons with the Tukey’s “Honest Significant Difference” method. **b** Stacked barcharts comparing the weekly relative abundance of bacterial families representing > 0.01 (1%) of the fecal swabs bacterial community at any time point within either T1 or T2. **c** Weekly beta-diversity of fecal swab microbiota communities between different microbiota input groups for both T1 and T2. Weekly population level PERMANOVA statistics (F.models and q-values) between microbiota input groups within the same trial and between trials within the same input group are detailed in Figure S3. Ellipses were generated around points to aid in visualizing group differences assuming a multivariate T-distribution with a 95% confidence interval. Fecal swabs were not collected for T1 CONV birds at week 6

Weekly fecal swabs were collected to assess microbiota shifts throughout both trials. Microbial community richness and evenness did not vary significantly by microbial input group, week, or trial according to the measured Shannon Index (*P* > 0.05) (Fig. 3a). Firmicutes were highly abundant for all three microbiota input groups in both trials (Figure S2). Lachnospiraceae was the most abundant family in the T1 CONV group feces across all time points (Fig. 3b). In T1 EGG group, Enterococcaceae was most abundant for the first 2 weeks, but shifted to a community more evenly split between Enterococcaceae and Lachnospiraceae for remaining weeks (Fig. 3b). Lachnospiraceae was most abundant in the feces for the first week in T1 ENV birds, with Peptostreptococcaceae becoming most abundant in later weeks (Fig. 3b). Similar to T1, the T2 CONV group had high abundances of Lachnospiraceae throughout the trial. Enterococcaceae was highly abundant in the EGG group feces throughout T2, excluding week 1, which had comparable abundances of Enterococcaceae and Enterobacteriaceae (Fig. 3b). While no family dominated at week 1, the T2 ENV group shifted to higher abundances of Peptostreptococcaceae from weeks 3 to 6. Relative abundance plots at the genus level are displayed in Supplemental Figure S2.

The community structure of fecal samples varied significantly by microbiota input source and trial according to the PERMANOVA (Fig. 3c, Figure S3). All comparisons between the 3 input groups were significantly different (q < 0.05) for both trials. Between trials, CONV and EGG groups were significantly different at all weeks tested (q < 0.05), though T1 and T2 ENV fecal communities were not significantly different at week 5 (q = 0.143). Differences in fecal community beta dispersion also existed. For example, the T1 ENV group was significantly more dispersed than the EGG group at week 1, and the CONV group at week 2 (*P* < 0.05) (Fig. 3a). The T2 ENV fecal communities were more dispersed than the EGG fecal communities at week 5 (*P* < 0.05) (Fig. 3a).

### GIT 16S rRNA gene analysis

#### Mucosa samples

Both lumen and mucosa samples were collected for the jejunum, ileum, and cecum. The mucosa and lumenal samples from the jejunum, ileum, and cecum had similar community structure initially but were significantly different from one another at later time points (Table S5). However, mucosa samples displayed similar trends to lumenal samples with regard to alpha and beta diversity when comparing between microbiota input groups and trials, so mucosal samples were analyzed but not included in the results outside of determining unique and shared ASVs for individual intestinal compartments. To be concise, only the lumenal samples are included in the results section. Plots characterizing the alpha and beta diversity of mucosal-associated microbial communities are in Supplemental Figure S4, S5, S10, S11, and S12. Supplemental Table S4 contains statistically significant differences between input groups and trials along the cecal mucosa.

#### Cecal lumen samples

**Fig. 4:**
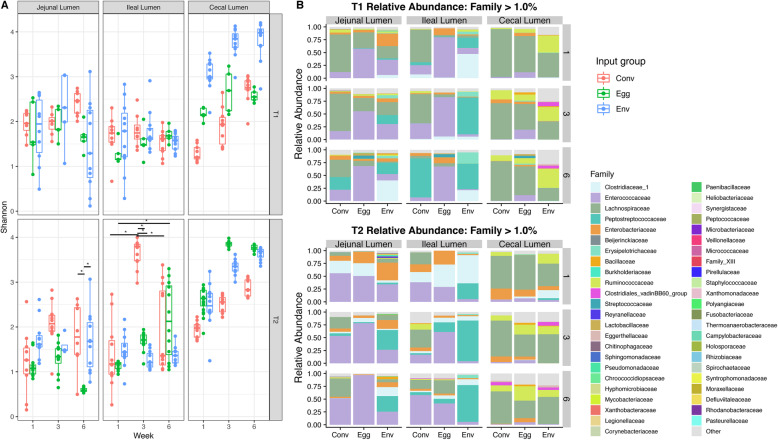
Comparisons of bacterial alpha diversity in the lumen of the jejunum, ileum, and cecum. **a** Changes in intestinal lumen microbiota alpha-diversity (Shannon index) for different microbiota input groups throughout the 6-week trial period for both T1 and T2. Comparisons between Shannon indices between microbiota input groups, weeks, and trials were made using an analysis of variance (ANOVA) followed by subsequent pairwise comparisons with the Tukey’s “Honest Significant Difference” method. Significance bars with an asterisk (*) indicate difference between microbiota input groups and/or time points (*P* < 0.05). **b** Stacked barcharts comparing the relative abundance of bacterial families representing > 0.01 (1%) of the lumenal bacterial community in any gut compartment within T1 or T2 at weeks 1, 3, and 6

Regardless of input group or trial, alpha diversity in the cecal lumen increased from weeks 1 to 6. The T1 EGG group was an exception and did not display significant weekly differences, suggesting no significant changes in community richness and evenness occurred (*P* > 0.05) (Fig. 4a, Table S4). Between input groups, T1 CONV cecal lumen samples displayed lower community richness and evenness than the ENV group at all weeks, and the EGG group at weeks 1 and 3 (*P* < 0.05) (Fig. 4a, Table S4). The T2 EGG group had significantly higher Shannon indices at weeks 3 and 6 in the cecal lumen (Fig. 4a, Table S3). Comparing cecal alpha diversity between trials, T2 CONV group had higher Shannon indices than T1 CONV group at weeks 1 and 3, and T1 ENV group displayed higher alpha diversity at weeks 1 and 3 than T2 ENV birds (Fig. 4a, Table S4). The T2 EGG group had significantly higher alpha diversity metrics at weeks 3 and 6 in the cecal lumen (Fig. 4a, Table S4).

Firmicutes comprised at least 78% of weekly cecal lumen samples for all microbiota inputs in both trials (Figure S4). Lachnospiraceae (37–94%) and, to a lesser extent, Ruminococcaceae (up to 51%), Enterobacteriaceae (up to 22%), Enterococcaceae (up to 19%), and Clostridiaceae 1 (up to 16%) formed a majority of the cecal microbial community at most time points (Fig. 4b). Relative abundance plots at the genus level are displayed in Supplemental Figure S5.

**Fig. 5:**
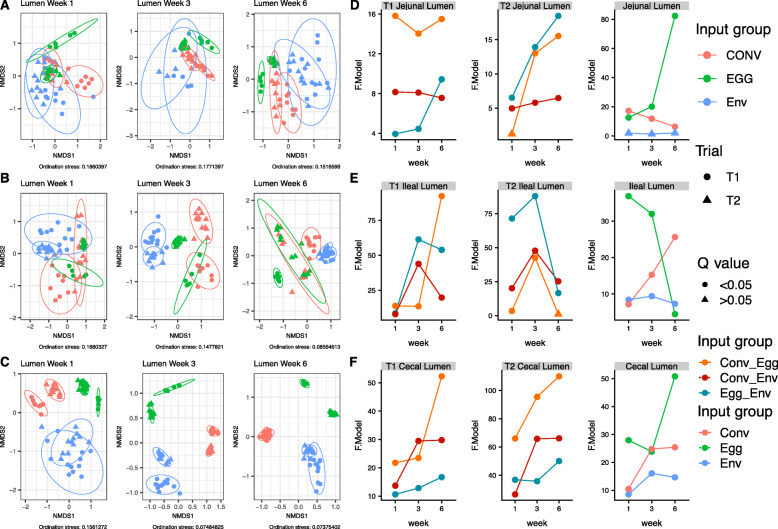
Beta-diversity of lumen bacterial communities between different microbiota input groups within the (**a**) jejunum, (**b**) ileum, and (**c**) cecum for both T1 and T2 and weeks 1, 3, and 6. Ellipses were generated around points to aid in visualizing group differences assuming a multivariate T-distribution with a 95% confidence interval. Population level PERMANOVA statistics (F.models and q-values) were also assessed between microbiota input groups within the same trial and between trials within the same input group for the (**d**) jejunum, (**e**) ileum, and (**f**) cecum

The different input sources altered microbial community compositions in the cecal lumen (Fig. 5c-f). Within trials, all the groups had significantly different community structures from one another at all weeks based on PERMANOVA (q < 0.05). Trial number also significantly altered the bacterial composition (q < 0.05). There were no statistical differences in dispersion between inputs or trials.

A core cecal microbiota was determined for each trial by identifying ASVs present in at least one of the input groups at a level of at least 10 reads (Figure S6). The T1 input groups shared a core of 25 ASVs in the cecum. Four ASVs were unique to the CONV and EGG groups while 40 ASVs were unique to the CONV and ENV groups. The core cecal microbiota of the T2 group was composed of 60 ASVs, of which 25 ASVs were unique to the CONV and EGG group, and 28 were unique to the CONV and ENV group. Nineteen ASVs composing > 1.0% of at least one of the input groups in both trials were identified. A majority of those ASVs belonged to Lachnospiraceae. A summary of highly abundant ASVs in each group is listed in Supplemental Figure S6.

#### Ileal lumen results

Ileal lumen community alpha diversity was not different (*P* > 0.05) between either the microbiota input group or the week for the T1 groups (Fig. 4a). The T2 groups displayed a similar trend as well, though the CONV input group had an ileal community with significantly higher Shannon index values at week 3 in the lumen, suggesting a more rich and evenly distributed microbial community (Fig. 4a). Firmicutes comprised 73–99% of the ileal lumen in both trials (Figure S4). Lachnospiraceae was 58–64% of the ileal lumen of T1 CONV group for the first 3 weeks of the trial before shifting to Peptostreptococcaceae (78%) by week 6 (Fig. 4b). Enterococcaceae composed the majority (68–79%) of the T1 EGG group ileal lumen community for the entirety of the study (Fig. 4b). Clostridiaceae_1 and Peptostreptococcaceae collectively composed 68% of the T1 ENV group ileal community at week 1 before shifting to a community structure mainly composed of Peptostreptococcaceae (54–73%) by week 3 (Fig. 4b). Enterococcaceae (39%), Lachnospiraceae (25%), Clostridiaceae_1 (20%), and Enterobacteriaceae (15%) comprised the T2 CONV ileal lumen at week 1, but Enterococcaceae increased to 59% of the bacterial community by week 6 (Fig. 4b). Similar to the T2 CONV group, the T2 EGG group had Clostridiaceae_1 (44%), Enterococcaceae (29%), and Enterobacteriaceae (27%) at week 1, with Enterococcaceae rising in abundance to 42% by week 6 (Fig. 4b). Peptostreptococcaceae (32%) and Clostridiaceae_1 (56%) were highly abundant in the T2 ENV group ileum at week 1 before shifting to a majority Peptostreptococcaceae by week 3 (81%), which remained dominant at week 6 (73%) (Fig. 4b). Relative abundance plots at the genus level are displayed in Supplemental Figure S5.

The T1 groups possessed a core ileal microbiota of 52 ASVs (Figure S7). There were an additional 14 ASVs that were unique to both the T1 CONV and EGG groups while 28 ASVs were unique to T1 CONV and ENV groups. The core ileal microbiota of the T2 groups was 110 ASVs (Figure S7). An additional 123 ASVs were unique to CONV and EGG groups, nearly triple the 34 ASVs exclusive to the T2 CONV and ENV groups. Fifteen ASVs were present at > 1.0% in at least one input group in either trial. The two most abundant ASVs, *Enterococcus* (ASV_1) and *Romboutsia* (ASV_2), were present in the ileum of all input groups for both trials but in different abundances (Figure S7).

Differences in ileal microbial community structure existed between trials and input groups (Fig. 5b-e). Within trials, input groups had significantly different microbiota compositions than one another at all weeks based on PERMANOVA (q < 0.05), with the exception of the T2 week 6 ileal lumen samples, where the CONV and EGG groups did not have statistically different bacterial communities (q = 0.269). Trial number also significantly altered the bacterial composition (q < 0.05). At week 1 the individual T1 EGG lumenal communities were significantly less dispersed than the ENV treatment (PERMDISP; *P* < 0.05), suggesting the bacterial communities of the individual EGG samples maintained a more similar structure to one another where the ENV group samples displayed a more disparate assemblage of bacterial communities. A similar trend was observed between the T2 EGG and CONV lumenal communities at week 1, with the T2 EGG bacterial communities significantly less dispersed than the bacterial communities in the T2 CONV group (*P* < 0.05). At week 6, the T2 ENV lumenal microbiota were less dispersed than the T2 EGG treatment (*P* < 0.05) (Fig. 5b-e).

#### Jejunum lumen results

The jejunum had no significant differences in Shannon diversity measurements by week or input source group in T1 (Fig. 4a). The T2 groups were, for the most part, stable across week and treatment as well, though the Shannon diversity measurements for the EGG group jejunal lumen community were significantly lower (*P* < 0.05) than the CONV and ENV group at week 6, suggesting the community did not become richer and more evenly distributed overtime (Fig. 4a).

Firmicutes dominated the jejunal community for all treatment groups and trials (Figure S4). Lachnospiraceae were highly abundant in the T1 CONV jejunal lumen at weeks 1 and 3 (74%) before splitting between Lachnospiraceae (40%), Peptostreptococcaceae (25%), and Enterococcaceae (22%) by week 6 (Fig. 4b). Enterococcaceae remained the most abundant fraction of the T1 EGG jejunal lumen (56–69%) for the duration of the trial (Fig. 4b). No single family dominated the T1 ENV jejunum, though Enterobacteriaceae (8–25%), Lachnospiraceae (26–31%), and Enterococcaceae (30%) were fairly abundant at earlier time points and Clostridiaceae_1 (40%) and Peptostreptococcaceae (18–23%) became more abundant at later ones (Fig. 4b). Enterococcaceae (56%) and, to a lesser extent, Clostridiaceae_1 (25%) made up a majority of the jejunal community in the T2 CONV group at week 1 (Fig. 4b). Lachnospiraceae (27–38%) replaced Clostridiaceae_1 as the second most abundant family for weeks 3 and 6, with Enterococcaceae (52–54%) remaining the most abundant during later time points. Similar to the T2 CONV group, Enterococcaceae (51%) and Clostridiaceae_1 (25%) were highly abundant in the T2 EGG jejunum at week 1, though Enterococcaceae (79%) quickly came to dominate by week 3 and remaining so at week 6 (98%) (Fig. 4b). Like the T1 ENV group, no single family dominated the T2 ENV jejunum, with Enterobacteriaceae (35%) and Enterococcaceae (34%) being fairly abundant at week 1 and Peptostreptococcaceae (31–39%) becoming more abundant at later weeks (Fig. 4b). Relative abundance plots at the genus level are displayed in Supplemental Figure S5.

The T1 jejunal core microbiota was composed of 85 ASVs shared between all input groups, with an additional 31 ASVs exclusive to the EGG and CONV groups while 26 ASVs were unique to the CONV and ENV groups (Figure S8). The T2 jejunal core microbiota was 131 ASVs with an additional 51 ASVs exclusive to the CONV and EGG groups and 57 ASVs exclusive to the ENV and CONV groups (Figure S8). There were 11 ASVs representing at least 1.0% of at least one of the microbiota input groups in both trials (Figure S8).

Jejunal beta diversity varied by trial and input source (Fig. 5a/d). PERMANOVA comparisons between the 3 input groups were significantly different (q < 0.05) in the jejunum with the exception of the week 1 lumenal samples of the T2 CONV and EGG groups (q = 0.269). Comparing beta diversity between trials, CONV and EGG input groups were significantly different in the lumen for all weeks (q < 0.05). T1 and T2 ENV groups were not significantly different in jejunal lumen community composition at weeks 1, 3, and 6 (q > 0.05). The T1 EGG group was less dispersed than the ENV group at week 6, and the T2 EGG group displayed less dispersion than the CONV and ENV groups at week 6 (*P* < 0.05) (Fig. 5a-d).

#### Similarities between trials

Trial 1 birds shared 118 ASVs between all input source groups, with an additional 33 being exclusive to EGG and CONV groups while 44 ASVs were unique to ENV and CONV groups (Figure S9). Trial 2 shared 225 ASVs between the input groups, with 120 ASVs being shared exclusively between the EGG and CONV groups, double the number shared between the CONV and ENV groups (60) (Figure S9). A core microbiota of 19 ASVs was shared between both the T1 and T2 birds. Of those, one strain of *Enterococcus* (ASV_1) composed between 7.76% and 38.58% of all the reads in all input groups for both trials (Figure S9). Strains of *Romboutsia* (ASV_2), *Clostridium* sensu stricto (ASV_5), *Escherichia* (ASV_6), and several unclassified ASVs belonging to the family Lachnospiraceae (ASV_3/4/7/8/14/19/21/30) were also highly abundant ASVs found in all input groups for both trials. Each trial possessed several unique strains of *Romboutsia* and unclassified Lachnospiraceae. A complete list of shared and unique ASVs shared is listed in Supplementary Figure S9.

#### SCFA results

**Fig. 6:**
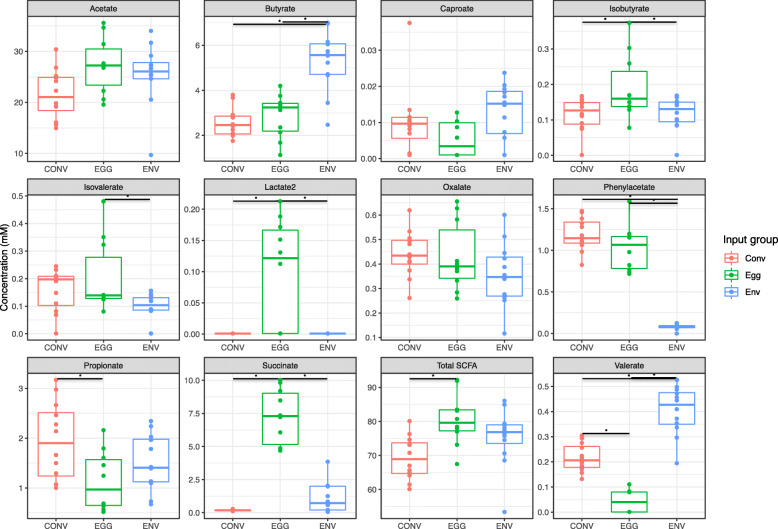
Concentrations (mM) of select short chain fatty acids in the cecum of each T2 microbiota input group at week 6. Comparisons between microbiota input groups were made using an analysis of variance (ANOVA) followed by subsequent pairwise comparisons with the Tukey’s “Honest Significant Difference” method. Significance bars with an asterisk (*) indicate significant differences between microbiota input groups (*P* < 0.05)

In order to assess the functionality of the microbes in the ceca for each input group, the SCFA profile of cecal contents from the T2 group at week 6 was determined (Fig. 6). Short-chain fatty acid analysis was not conducted for T1 birds. Cecal contents from the EGG group had significantly reduced propionate, and increased isobutyrate and succinate concentrations compared to the CONV group (*P* < 0.05). In contrast, cecal contents from the ENV group displayed increased butyrate and decreased phenylacetate production when compared to the CONV group (*P* < 0.05). Interestingly, lactate_2 was only detected in the cecal contents from the EGG group, and was not found in either the CONV or ENV groups. The EGG group had the highest overall SCFA concentration out of all input groups.

## Discussion

Acquisition and succession of host GIT microbiota has important implications on the breakdown of foodstuffs, nutrient absorption, and host health [1–3]. Therefore, it is essential to understand the sources from which hosts acquire their microbiota in order to identify intervention points for raising healthier, more productive chickens. In mammals, postnatal maternal contact is an important source of microbiota for establishment of commensal organisms in the GIT of offspring, a source absent in modern poultry hatcheries [6, 7, 14]. Due to separation, a majority of the microbiota in poultry may be acquired through the external environment, although some *in ovo* transfer of microbes is possible [13, 15, 20]. The impact of the eggshell microbiota as a microbiota input source that drives succession within the chicken GIT is currently unknown. Here, we assessed the succession and functionality of GIT microbiota in recently hatched chicks exclusively exposed to two different microbiota input sources, either eggshell (EGG) or environmental (ENV) microbiota and compare those communities to the GIT microbiota of “conventional” chicks (CONV) that were exposed to both.

During oviposition, eggs pass through the lower portion of the intestinal tract and exit through the cloaca, coating the eggshell with fecal microbiota from the hen [24]. Many bacteria shed in feces are capable of forming spores, suggesting the hen fecal material that coats the outside of recently laid eggs could harbor viable anaerobes, or spores of the anaerobes, that could be ingested upon hatch by chicks and initiate GIT microbiota succession [26]. Additionally, transmission of spore-forming microbes via the eggshell to newly hatched chickens through spray application of adult cecal contents to incubating eggs has recently been observed, suggesting spore-formers on the eggshell surface are successfully ingested and establish within the GIT [37]. We cultured viable, anaerobic bacteria from chicken eggshells after the 21-day incubation period. Microbiota sequence analysis from eggshells revealed bacterial communities that were diverse, with high abundances of common intestinal commensals belonging to Lactobacillaceae, Lachnospiraceae, and Enterococcaceae [2, 18, 38–40]. Early exposure to a diverse microbiota, similar to that detected on the eggshell, is critical for the establishment of a healthy intestinal community in avian species post-hatch [41]. Over 50 ASVs were shared between the eggshells in both trials, suggesting the presence of a core eggshell microbiota that could be passed from hen to chick via the eggshell. The presence of culturable organisms on the eggshell surface indicates it is an early inoculum for hatching chicks, allowing for vertical transmission of commensals from hen to chick. Many conventional hatcheries use egg washing, and while washing may prevent pathogen colonization, it may inadvertently prevent acquisition of maternal commensals.

Microbiota input source had a major impact on the bacterial community structure, composition, and succession within the different gut compartments assessed in this study. This was especially true in the ceca, which were highly segregated based on microbiota input source and trial. Input groups, with the exception of the T1 EGG group, displayed increasing richness and evenness of the cecal community as time went on. Increasing alpha diversity has been observed in other longitudinal studies of the chicken ceca, suggesting successional processes were occurring in all input groups [39, 42, 43]. Lachnospiraceae was the most abundant family in the ceca for both trials, regardless of input group or time point, though Ruminococcaceae, Enterobacteriaceae, Enterococcaceae, and Clostridiaceae_1 were also identified, an observation in accordance with previous studies [39, 44]. The CONV birds in both trials were characterized by high initial abundances of Lachnospiraceae, with Ruminococcaceae and Clostridiaceae increasing as time went on. This successional pattern was in line with other studies of the chicken cecum [2, 18, 39]. Though sharing taxa, community compositions and successional patterns for input groups remained unique, resulting in altered microbiota structures. Differences in cecal community structure also displayed different functional capabilities as measured by SCFA production, which can serve as a proxy for overall functionality in a bacterial community [45]. Differences in cecal SCFA production could produce impactful changes for susceptibility to infectious diseases, intestinal health, and feed efficiency [4, 46, 47]. Although sharing taxa, the cecal microbial community comparisons and functional differences suggested the microbiota in this gut compartment couldn’t be exclusively attributed to either the eggshell or the environment. The ultimate community structure in the chicken ceca is likely a complex and interdependent interplay between the two microbiota sources, with the absence of one microbiota source or the other leading to significant shifts in the successional process that require further investigation.

Microbiota input sources also affected the bacterial communities in the small intestine. Like the ceca, jejunal and ileal microbial communities tended to segregate based on input source. Small intestinal bacterial communities were more stable in richness and evenness when compared to the cecal communities both on a week-to-week basis and between the input groups and trials, a phenomenon observed in previous studies [39]. The succession pattern in the ileum and jejunum for the EGG group (both trials) and the T2 CONV seem to align with a recent study, with high levels of Enterococcaceae strains initially and increases to strains associated with Lachnospiraceae, Peptostreptococcaceae, and Ruminococcaceae occurring late in succession [39]. However this previous study, and others, identified *Lactobacillus* (family: Lactobacillaceae) as a major component of the upper intestinal tract microbiota, a trend we failed to replicate in either of our trials, though Lactobacillaceae were a highly abundant community member on eggshells throughout incubation [20, 39]. Comparisons of the bacterial community structure in the small intestine showed similarity between the T2 EGG and CONV groups in the jejunum at week 1 and the ileum at week 6. These data suggest eggshell microbiota may play an important role in the establishment, maturation, and community structure of the small intestine, where the eggshell microbial community may contain founder species that modify the environment to mediate “conventional” successional processes [25]. These observations warrant further study of the microbiota associated with both the eggshell and the small intestine, especially in the hours and days immediately post-hatch. The small intestine also displayed a high number of “core” ASVs, where the lowest number of conserved ASVs between the input groups (trial 1 ileum) was over fifty, double the number of conserved ASVs found in the T1 ceca. This suggests segments of the small intestine select for specific taxa.

While providing an overview of the potential ASVs present in the GIT and a general sense of which taxa may be highly abundant at a given time, the fecal microbiota was a weak proxy for the community structure for any one gut compartment in particular. Fecal communities sometimes resembled those in the ceca more closely, sometimes the ileum or jejunum, and sometimes fecal microbiota did not resemble any other compartment in particular. The unrelatedness of fecal swabs to the actual community composition within individual intestinal segments agreed with similar findings of other chicken microbiota studies [48, 49]. With the above caveats in mind, the successional pattern in the feces, specifically the ENV group of both trials, did appear to match previously established successional patterns, with rapid colonizers like Clostridiaceae, Enterobacteriaceae, and Lachnospiraceae being abundant early in succession before a shift toward increasing Peptostreptococcaceae at later time points [10].

Several of the core taxa identified in this study are currently being studied as important modulators of host physiology. *Turicibacter* (0.02–5.50% total reads/input group) is a highly heritable genus of bacteria that is able to interact with host-derived bile acids and play a role in the neurotransmitter regulation in the mammalian gut [50, 51]. *Romboutsia* (1.15–19.34% total reads/input group) appeared as the second most highly abundant genera throughout the study, and was identified as a member of the core microbiota in both trials. *Romboutsia* is commonly associated with the intestinal tract of mammalian species and has only recently been identified in poultry [17, 39, 52, 53]. Both *Turicibacter* and *Romboutsia* have recently been implicated in metabolic alterations in the serum and hippocampus of rats, suggesting these genera may be important in the regulation of host energy metabolism through host-microbial cross-talk via regulation of neurotransmitter levels [54]. The thin mucosal layer and prevalent innervation via the enteric nervous system make the small intestine an ideal location for host-microbiota interactions that impact hormone levels, metabolism, and other important physiological processes [55, 56]. The abundance of *Turicibacter* and *Romboutsia* in the small intestine and their potential roles in hormonal and metabolic regulation of the host makes additional work characterizing both *Turicibacter* and *Romboutsia* important for furthering understanding of modulation of host health and productivity by specific taxa along the intestinal tract. It is likely other bacteria in the small intestine have similar impacts as well, providing potential targets for interventions in many different livestock animals, including chickens. This highlights the importance of tracking microbiota shifts in the small intestine during poultry studies. Multiple abundant ASVs identified as core microbiota were uncharacterized genera within the family Lachnospiraceae, emphasizing the importance of phenotypically characterizing novel members of the poultry microbiota.

Replicate trials with independent bird flocks allowed for the identification of conserved taxa and trends, such as the relative consistency of the successional patterns in the small intestine for both the EGG and ENV groups and the cecum of the CONV groups. Two animal trials also allowed us to identify discrepancies between replications, such as the shift in the T1 CONV ileum toward a community characterized by Peptostreptococcaceae while the T2 CONV ileum shifted to an Enterococcaceae-centric community. The strengths provided through independent replication and the sampling of different intestinal compartments in successional studies exemplified the importance of including both replication and multi-compartment sampling into future poultry microbiota analyses, preferably with birds originating from different hatcheries, breeds, and/or production systems. The fact that the core microbiota identified is shared between two unrelated flocks increases the strength of the observations from this study. It is possible the eggshell culturing methods described here may not have fully captured the total viable microbial populations on the eggshell and further work with different culturing methods may aid in the full elucidation of the viable microbial community on the eggshell. It is also possible the vertical inheritance we attributed to the eggshell may be partially explained by *in ovo* transfer of maternal microbes. Several studies suggest the embryo itself may harbor maternal microbes that colonize the embryo during the egg formation process [20, 57]. However, our previous hatching of germ-free turkey poults in an unrelated study suggests *in ovo* transmission of microbes may not be very prevalent [27].

## Conclusions

Distinct microbial communities and differing functional profiles (SCFAs) were observed between birds exposed to the eggshell microbes only, environmental microbes only, or both (conventionally reared). While differences were observed between egg sources, the main driver of differences in the chicken intestinal tract was exposure to the eggshell versus environmental microbiota. Our data suggested the eggshell played an appreciable role in the development of the chicken intestinal microbiota, especially in the small intestine where the community structure of the eggshell exposed-only birds was found to be similar to the structure of the conventionally hatched and raised birds. Overall, our data identify a complex interplay between the eggshell and environmental microbiota during establishment and succession within the chicken gut. Further studies should be conducted to explore the eggshell as a progenitor of physiologically important, early colonizing bacteria and how this eggshell microbial community can be modified as a potential intervention point to alter successional processes in the gut to improve GIT function and prevent pathogen carriage.

## Supplementary information

**Additional file 1: Table S1.** Study design and sample collection for both T1 and T2. Samples collected at each time point for each input group in each treatment are noted.

**Additional file 2: Table S2.** Core amplicon sequence variants (ASVs) from the T1 and T2 eggshells. Only ASVs representing > 0.1% abundance are included. “Shared Eggshell ASVs” were present at > 0.1% abundance in both T1 and T2.

**Additional file 3: Table S3.** PERMANOVA and beta dispersion results for eggshell microbiota community beta-diversity comparisons for both T1 and T2. Bold values represent statistical significance as determined by PERMANOVA (q < 0.05) or beta dispersion (*P* < 0.05).

**Additional file 4: Table S4.** Significant alpha diversity comparison results for the lumen- and mucosa-associated microbial communities in the cecum. Comparisons are labeled input group_week_trial-input group_week_trial. Comparisons between microbiota input groups, trials, and weeks were made using an analysis of variance (ANOVA) followed by subsequent pairwise comparisons with the Tukey’s “Honest Significant Difference” method. Only statistically significant comparisons are included in the table (*P* < 0.05).

**Additional file 5: Table S5.** Comparisons of beta diversity (PERMANOVA) for the lumen- and mucosa-associated microbial communities at weeks 1, 3, and 6 for T1 and T2 birds. Only statistically significant comparisons are included in the Table (*P <* 0.05). Bold values represent statistical significance as determined by PERMANOVA or beta dispersion (*P* < 0.05).

**Additional file 6: Figure S1.** Stacked barcharts comparing the weekly relative abundance of bacterial phyla (> 0.1%) and genera (> 2.0%) of the eggshell bacterial community at any time point within T1 and T2.

**Additional file 7: Figure S2.** Stacked barcharts comparing the weekly relative abundance of bacterial phyla (> 1.0%) and genera (> 2.0%) of the fecal swab bacterial community for T1 and T2.

**Additional file 8: Figure S3.** Weekly population level PERMANOVA statistics (F.models and q-values) for the fecal swabs between microbiota input groups within the same trial and between trials within the same input group.

**Additional file 9: Figure S4.** Stacked barcharts comparing the relative abundance of bacterial phyla (> 1.0%) along the intestinal tract, both lumen and mucosa, at weeks 1, 3, and 6 for T1 and T2 birds.

**Additional file 10: Figure S5.** Stacked barcharts comparing the relative abundance of bacterial genera (> 2.0%) along the intestinal tract, both lumen and mucosa, at weeks 1, 3, and 6 for T1 and T2 birds.

**Additional file 11: Figure S6.** List of cecum-associated bacterial ASVs shared between bacterial input groups and trials. ASVs are listed with a number followed by the associated family and genus classifications and a number if there are multiple, unique ASVs with the same family and genus classifications. Bolded numbers are > 1.0% relative abundance. Core shared ASVs in the upper table are those present at > 1.0% relative abundance in a least one of the microbial input groups in both trials. ASVs present at > 1.0% relative abundance in a least one of the microbial input groups in only one trial are found in the lower potion of the table. Venn diagrams at the bottom of the figure show the sharing of ASVs between microbial input groups with > 10 reads within a trial.

**Additional file 12: Figure S7.** List of ileum-associated bacterial ASVs shared between bacterial input groups and trials. ASVs are listed with a number followed by the associated family and genus classifications and a number if there are multiple, unique ASVs with the same family and genus classifications. Bolded numbers are > 1.0% relative abundance. Core shared ASVs in the upper table are those present at > 1.0% relative abundance in a least one of the microbial input groups in both trials. ASVs present at > 1.0% relative abundance in a least one of the microbial input groups in only one trial are found in the lower potion of the table. Venn diagrams at the bottom of the figure show the sharing of ASVs between microbial input groups with > 10 reads within a trial.

**Additional file 13: Figure S8.** List of jejunum-associated bacterial ASVs shared between bacterial input groups and trials. ASVs are listed with a number followed by the associated family and genus classifications and a number if there are multiple, unique ASVs with the same family and genus classifications. Bolded numbers are > 1.0% relative abundance. Core shared ASVs in the upper table are those present at > 1.0% relative abundance in a least one of the microbial input groups in both trials. ASVs present at > 1.0% relative abundance in a least one of the microbial input groups in only one trial are found in the lower potion of the table. Venn diagrams at the bottom of the figure show the sharing of ASVs between microbial input groups with > 10 reads within a trial.

**Additional file 14: Figure S9.** List of all bacterial ASVs shared between bacterial input groups and trials. ASVs are listed with a number followed by the associated family and genus classifications and a number if there are multiple, unique ASVs with the same family and genus classifications. Bolded numbers are > 1.0% relative abundance. Core shared ASVs in the upper table are the core ASVs shared between both trials present at > 1.0% relative abundance in a least one of the microbial input groups in both trials and present at > 10 reads in all the microbial input groups in both trials. ASVs present at > 1.0% relative abundance in a least one of the microbial input groups and present at > 10 reads in all the microbial input groups within only one trial are found in the lower potion of the table. Venn diagrams at the bottom of the figure show the sharing of ASVs between microbial input groups with > 10 reads within a trial.

**Additional file 15: Figure S10.** Changes in mucosal microbiota alpha-diversity (Shannon index) for different microbiota input groups throughout the 6-week trial period for both T1 and T2. Comparisons between Shannon indices between microbiota input groups, weeks, and trials were made using an analysis of variance (ANOVA) followed by subsequent pairwise comparisons with the Tukey’s “Honest Significant Difference” method. Significance bars with an asterisk (*) indicate difference between microbiota input groups and/or time points (*P* < 0.05).

**Additional file 16: Figure S11.** Stacked barcharts comparing the relative abundance of bacterial families (> 1.0%) along the intestinal mucosa at weeks 1, 3, and 6 for T1 and T2 birds.

**Additional file 17: Figure S12.** Beta-diversity of mucosal bacterial communities between different microbiota input groups within the (A) jejunum, (B) ileum, and (C) cecum for both T1 and T2 at weeks 1, 3, and 6. Ellipses were generated around points to aid in visualizing group differences assuming a multivariate T-distribution with a 95% confidence interval. Population level PERMANOVA statistics (F.models and q-values) were also assessed between microbiota input groups within the same trial and between trials within the same input group for the (D) jejunum, (E) ileum, and (F) cecum.

## Data Availability

All raw 16S rRNA gene sequencing data and associated metadata used in this study was deposited in the National Center for Biotechnology Information (NCBI) Sequence Read Archive (SRA) database under the BioProject number PRJNA607089.

## Abbreviations

16S rRNA: 16S ribosomal ribonucleic acid
ANOVA: Analysis of variance
ASV: Amplicon sequence variant
BHI: Brain heart infusion
CFU: Colony-forming unit
GIT: Gastrointestinal tract
NMDS: Non-metric multidimensional scaling
PBS: Phosphate-buffered saline
PERMANOVA: Permutational multivariate analysis of variance
PERMDISP: Permutation multivariate analysis of dispersion
SCFA: Short chain fatty acid
