# Supplementary material for: Eggshell and environmental bacteria contribute to the intestinal microbiota of growing chickens
Source: J Anim Sci Biotechnol. 2020 Jun 11;11:60. doi: 10.1186/s40104-020-00459-w (PMC7288515; doi:10.1186/s40104-020-00459-w)
Supplement: Supplementary file 1 — Additional file 1: Table S1. Study design and sample collection for both T1 and T2. Samples collected at each time point for each input group in each treatment are noted. [file 40104_2020_459_MOESM1_ESM.pdf]

Timepoint	Day	-21 (Eggs begin Incubation)	-14	-7	0 (Hatch)	7	14	21	28	35	42
T1 Sampling	CONV	7 eggs (swab)	7 eggs (swab)	7 eggs (swab)	7 eggs (swab)<br>30 birds hatch<br>(10 legband)	9 Necro<br>(10 Fecals)	(10 Fecals)	10 Necro<br>(10 Fecals)	(10 Fecals)	(10 Fecals)	11 Necro
	EGG	–	–	–	15 birds hatch<br>(5 legband)	5 Necro<br>(10 Fecal)	(5 Fecal)	5 Necro<br>(10 Fecals)	(5 Fecals)	(5 Fecals)	5 Necro<br>(5 Fecals)
	ENV	–	–	–	36 birds hatch<br>(12 legband)	12 Necro<br>(12 Fecal)	(12 Fecal)	12 Necro<br>(12 Fecal)	(12 Fecal)	(12 Fecal)	12 Necro<br>(12 Fecal)
T2 Sampling	CONV	5 eggs (wash)	5 eggs (wash)	5 eggs (wash)	10 eggs (wash: 5 pre-hatch/5 post-hatch)<br>36 birds hatch<br>(12 legband)	12 Necro<br>(12 Fecal)	(12 Fecal)	12 Necro<br>(12 Fecal)	(12 Fecal)	(12 Fecal)	12 Necro<br>(12 Fecal)
	EGG	–	–	–	51 birds hatch	13 Necro 14 Birds Cecal Contents Only<br>(12 Fecal)	(12 Fecal)	12 Necro<br>(12 Fecal)	(12 Fecal)	(12 Fecal)	12 Necro<br>(12 Fecal)
	ENV	–	–	–	35 birds hatch	12 Necro<br>(12 Fecal)	(12 Fecal)	11 Necro<br>(12 Fecal)	(12 Fecal)	(12 Fecal)	12 Necro<br>(12 Fecal)
