# Supplementary material for: Eggshell and environmental bacteria contribute to the intestinal microbiota of growing chickens
Source: J Anim Sci Biotechnol. 2020 Jun 11;11:60. doi: 10.1186/s40104-020-00459-w (PMC7288515; doi:10.1186/s40104-020-00459-w)
Supplement: Supplementary file 2 — Additional file 2: Table S2. Core amplicon sequence variants (ASVs) from the T1 and T2 eggshells. Only ASVs representing > 0.1% abundance are included. “Shared Eggshell ASVs” were present at > 0.1% abundance in both T1 and T2. [file 40104_2020_459_MOESM2_ESM.pdf]

T2 Eggshell-Specific ASVs (Mean Abundance)	
ASV_181_Enterococcaceae_Enterococcus	**3.53**
ASV_167_Streptococcaceae_Streptococcus	**3.31**
ASV_265_Lactobacillaceae_Lactobacillus	**1.47**
ASV_176_Corynebacteriaceae_Corynebacterium_1	**1.32**
ASV_304_Staphylococcaceae_Staphylococcus	**1.12**
ASV_199_Bacteroidaceae_Bacteroides	**1.09**
ASV_340_Prevotellaceae_Prevotellaceae_Ga6A1_group	0.92
ASV_349_Fusobacteriaceae_Fusobacterium	0.89
ASV_247_Bacteroidaceae_Bacteroides	0.76
ASV_331_Atopobiaceae_Olsenella	0.71
ASV_365_Lactobacillaceae_Lactobacillus	0.68
ASV_369_Staphylococcaceae_Jeotgalicoccus	0.66
ASV_362_Aerococcaceae_Aerococcus	0.64
ASV_317_Bacteroidaceae_Bacteroides	0.64
ASV_364_Aerococcaceae_Facklamia	0.62
ASV_358_Actinomycetaceae_Actinomyces	0.59
ASV_381_Lactobacillaceae_Lactobacillus	0.57
ASV_334_Bacteroidaceae_Bacteroides	0.45
ASV_391_Bacteroidaceae_Bacteroides	0.42
ASV_449_Staphylococcaceae_Jeotgalicoccus	0.39
ASV_28_Lachnospiraceae_NA	0.37
ASV_471_Corynebacteriaceae_Corynebacterium_1	0.33
ASV_437_Rikenellaceae_NA	0.33
ASV_502_Moraxellaceae_Psychrobacter	0.31
ASV_345_Prevotellaceae_Prevotellaceae_UCG-001	0.31
ASV_335_Bacteroidaceae_Bacteroides	0.29
ASV_408_Rikenellaceae_Rikenellaceae_RC9_gut_group	0.28
ASV_214_Ruminococcaceae_Flavonifractor	0.26
ASV_485_Bacteroidaceae_Bacteroides	0.25
ASV_252_Prevotellaceae_Prevotellaceae_UCG-001	0.23
ASV_3_Lachnospiraceae_NA	0.23
ASV_530_Enterococcaceae_NA	0.22
ASV_612_Bacteroidaceae_Bacteroides	0.21
ASV_505_Lactobacillaceae_Lactobacillus	0.21
ASV_70_Clostridiaceae_1_Clostridium_sensu_stricto_1	0.20
ASV_691_Bacteroidaceae_Bacteroides	0.20
ASV_450_Bacteroidaceae_Bacteroides	0.19
ASV_601_Aeromonadaceae_Oceanisphaera	0.17
ASV_31_Ruminococcaceae_Subdoligranulum	0.17
ASV_38_Lachnospiraceae_Sellimonas	0.14
ASV_555_Lactobacillaceae_Lactobacillus	0.14
ASV_616_Ruminococcaceae_Ruminococcaceae_UCG-005	0.14
ASV_571_Ruminococcaceae_Ruminococcaceae_UCG-007	0.13
ASV_723_Muribaculaceae_NA	0.13
ASV_377_Marinifilaceae_Odoribacter	0.13
ASV_671_Carnobacteriaceae_Atopostipes	0.13
ASV_542_Prevotellaceae_NA	0.13
ASV_746_Bacteroidaceae_Bacteroides	0.13
ASV_210_Lachnospiraceae_Lachnoclostridium	0.12
ASV_628_Bacteroidaceae_Bacteroides	0.12
ASV_635_Bacteroidaceae_Bacteroides	0.12
ASV_136_Lachnospiraceae_NA	0.12
ASV_430_Ruminococcaceae_Ruminococcaceae_UCG-008	0.12
ASV_722_Prevotellaceae_Prevotellaceae_Ga6A1_group	0.11
ASV_380_Synergistaceae_Synergistes	0.11
ASV_869_Aeromonadaceae_Oceanisphaera	0.11
ASV_694_Micrococcaceae_Yaniella	0.10
ASV_719_Ruminococcaceae_Subdoligranulum	0.10
ASV_155_Peptostreptococcaceae_Romboutsia	0.10

Shared Eggshell ASVs (T2 Abundance/T1 Abundance)		
ASV_103_Lactobacillaceae_Lactobacillus	**7.29**	**1.16**
ASV_2_Peptostreptococcaceae_Romboutsia	**5.42**	**3.27**
ASV_127_Lactobacillaceae_Lactobacillus	**5.00**	0.48
ASV_6_Enterobacteriaceae_Escherichia/Shigella	**4.75**	**10.33**
ASV_125_Lactobacillaceae_Lactobacillus	**3.56**	**3.21**
ASV_175_Lactobacillaceae_Lactobacillus	**3.04**	0.58
ASV_160_Lactobacillaceae_Lactobacillus	**3.00**	0.91
ASV_165_Veillonellaceae_Megamonas	**2.32**	0.41
ASV_8_Lachnospiraceae_NA	**1.67**	**1.94**
ASV_151_Veillonellaceae_Megamonas	**1.59**	**4.30**
ASV_184_Veillonellaceae_Megamonas	**1.48**	0.47
ASV_166_Acidaminococcaceae_Phascolarctobacterium	**1.24**	**1.21**
ASV_207_Lactobacillaceae_Lactobacillus	**1.06**	**1.73**
ASV_301_Lactobacillaceae_Lactobacillus	0.93	0.21
ASV_1_Enterococcaceae_Enterococcus	0.91	**1.75**
ASV_323_Lactobacillaceae_Lactobacillus	0.85	0.26
ASV_298_Lactobacillaceae_Lactobacillus	0.74	0.51
ASV_94_Ruminococcaceae_Faecalibacterium	0.68	0.54
ASV_150_Corynebacteriaceae_Corynebacterium_1	0.63	**1.14**
ASV_237_Bacteroidaceae_Bacteroides	0.63	**1.08**
ASV_208_Bacteroidaceae_Bacteroides	0.61	**2.53**
ASV_200_Lachnospiraceae_NA	0.53	0.15
ASV_264_Atopobiaceae_Olsenella	0.48	0.83
ASV_11_Clostridiaceae_1_Clostridium_sensu_stricto_1	0.48	0.42
ASV_33_Lachnospiraceae_NA	0.41	0.55
ASV_135_Ruminococcaceae_Faecalibacterium	0.40	0.81
ASV_238_Veillonellaceae_Megasphaera	0.38	0.15
ASV_232_Lachnospiraceae_Blautia	0.37	0.14
ASV_261_Bacteroidaceae_Bacteroides	0.35	0.37
ASV_81_Lachnospiraceae_CHKCI001	0.31	0.27
ASV_277_Bacteroidaceae_Bacteroides	0.30	**1.15**
ASV_65_Lachnospiraceae_Sellimonas	0.29	0.83
ASV_13_Erysipelotrichaceae_Turicibacter	0.29	0.91
ASV_4_Lachnospiraceae_NA	0.27	**2.74**
ASV_139_Bacillaceae_Bacillus	0.26	0.47
ASV_382_Desulfovibrionaceae_Desulfovibrio	0.24	0.45
ASV_19_Lachnospiraceae_NA	0.24	**1.06**
ASV_410_Tannerellaceae_Parabacteroides	0.22	0.17
ASV_443_Peptococcaceae_Peptococcus	0.22	0.14
ASV_36_Lachnospiraceae_NA	0.22	**2.00**
ASV_23_Peptostreptococcaceae_Romboutsia	0.22	0.10
ASV_114_Lachnospiraceae_Anaerosporobacter	0.20	**2.22**
ASV_122_Lachnospiraceae_NA	0.20	0.13
ASV_368_Veillonellaceae_Megamonas	0.20	0.36
ASV_383_Bifidobacteriaceae_Bifidobacterium	0.16	0.41
ASV_14_Lachnospiraceae_NA	0.16	**1.18**
ASV_110_Lachnospiraceae_Sellimonas	0.15	0.94
ASV_318_Prevotellaceae_Prevotellaceae_UCG-001	0.15	0.57
ASV_243_Muribaculaceae_NA	0.12	0.34
ASV_540_Lactobacillaceae_Lactobacillus	0.12	0.25
ASV_617_Prevotellaceae_Alloprevotella	0.11	0.26
ASV_607_Ruminococcaceae_NA	0.10	0.17
ASV_142_Lachnospiraceae_CAG-56	0.10	0.71
ASV_43_Ruminococcaceae_Butyricicoccus	0.10	0.53
ASV_61_Erysipelotrichaceae_Erysipelatoclostridium	0.10	0.44

T1 Eggshell-Specific ASVs (Mean Abundance)	
ASV_24_Bacillaceae_NA	**2.50**
ASV_10_Enterobacteriaceae_Klebsiella	**1.82**
ASV_86_Lachnospiraceae_NA	**1.47**
ASV_270_Lactobacillaceae_Lactobacillus	**1.44**
ASV_133_Clostridiaceae_1_Clostridium_sensu_stricto_1	**1.35**
ASV_186_Veillonellaceae_Megamonas	0.68
ASV_112_Ruminococcaceae_Pseudoflavonifractor	0.50
ASV_493_Lactobacillaceae_Lactobacillus	0.49
ASV_163_Ruminococcaceae_Ruminiclostridium_9	0.46
ASV_444_Helicobacteraceae_Helicobacter	0.45
ASV_539_Deinococcaceae_Deinococcus	0.44
ASV_388_Pseudomonadaceae_Pseudomonas	0.42
ASV_339_Bacteroidaceae_Bacteroides	0.40
ASV_84_Moraxellaceae_Acinetobacter	0.40
ASV_20_Lachnospiraceae_Eisenbergiella	0.40
ASV_262_Bacteroidaceae_Bacteroides	0.39
ASV_303_Prevotellaceae_Alloprevotella	0.38
ASV_600_Family_XI_Murdochiella	0.34
ASV_574_Family_XI_Peptoniphilus	0.32
ASV_537_Family_XI_Gallicola	0.31
ASV_195_Xanthomonadaceae_Stenotrophomonas	0.30
ASV_98_Erysipelotrichaceae_Erysipelatoclostridium	0.29
ASV_512_Deferribacteraceae_Mucispirillum	0.27
ASV_62_Lachnospiraceae_Shuttleworthia	0.27
ASV_427_Prevotellaceae_Prevotellaceae_UCG-001	0.27
ASV_622_Prevotellaceae_Prevotellaceae_UCG-001	0.26
ASV_32_Lachnospiraceae_NA	0.25
ASV_548_Bifidobacteriaceae_Aeriscardovia	0.21
ASV_740_Prevotellaceae_Prevotellaceae_UCG-001	0.20
ASV_144_Peptostreptococcaceae_Romboutsia	0.20
ASV_473_Bacteroidaceae_Bacteroides	0.19
ASV_85_Staphylococcaceae_Staphylococcus	0.19
ASV_385_Lachnospiraceae_NA	0.18
ASV_183_Erysipelotrichaceae_Erysipelatoclostridium	0.18
ASV_791_Moraxellaceae_Enhydrobacter	0.17
ASV_654_Muribaculaceae_NA	0.15
ASV_209_Lachnospiraceae_NA	0.15
ASV_795_Rhizobiaceae_NA	0.15
ASV_352_Lachnospiraceae_Sellimonas	0.14
ASV_718_Lactobacillaceae_Lactobacillus	0.14
ASV_586_Ruminococcaceae_Subdoligranulum	0.14
ASV_300_Staphylococcaceae_Staphylococcus	0.14
ASV_866_Micrococcaceae_Rothia	0.14
ASV_615_Ruminococcaceae_Fournierella	0.14
ASV_544_Coriobacteriaceae_Enorma	0.13
ASV_547_Chitinophagaceae_Hydrotalea	0.13
ASV_963_Muribaculaceae_NA	0.12
ASV_152_Ruminococcaceae_NA	0.12
ASV_40_Lachnospiraceae_Sellimonas	0.12
ASV_68_Staphylococcaceae_Staphylococcus	0.11
