# Supplementary material for: Eggshell and environmental bacteria contribute to the intestinal microbiota of growing chickens
Source: J Anim Sci Biotechnol. 2020 Jun 11;11:60. doi: 10.1186/s40104-020-00459-w (PMC7288515; doi:10.1186/s40104-020-00459-w)
Supplement: Supplementary file 3 — Additional file 3: Table S3. PERMANOVA and beta dispersion results for eggshell microbiota community beta-diversity comparisons for both T1 and T2. Bold values represent statistical significance as determined by PERMANOVA (q < 0.05) or beta dispersion (P < 0.05). [file 40104_2020_459_MOESM3_ESM.pdf]

	Comparison	F.Model	R2	p.fdr (Adonis)	p.adj (Beta Disp)
ISU vs MMM					
	Wk0 T1 vs Wk0 T2	5.800	0.367	**0.013**	0.984
	Wk1 T1 vs Wk1 T2	3.922	0.304	**0.013**	0.549
	Wk2 T1 vs Wk2 T2	4.989	0.384	**0.014**	0.178
	Wk3 T1 vs Wk3 T2 (Clean)	3.067	0.254	**0.014**	0.999
	Wk3 T1 vs Wk3 T2 (Dirty)	2.975	0.248	**0.014**	0.593
Weekly comparisons					
	T1 Wk0 vs Wk1	1.197	0.098	0.285	0.976
	T1 Wk0 vs Wk2	3.161	0.240	**0.015**	0.967
	T1 Wk0 vs Wk3	2.033	0.156	0.055	0.632
	T1 Wk1 vs Wk2	1.443	0.138	0.131	1.000
	T1 Wk1 vs Wk3	0.840	0.078	0.645	0.997
	T1 Wk2 vs Wk3	0.876	0.089	0.599	0.999
	T2 Wk0 vs Wk1	0.993	0.110	0.483	1.000
	T2 Wk0 vs Wk2	1.348	0.144	0.122	0.998
	T2 Wk0 vs Wk3 (Clean)	3.571	0.309	**0.015**	0.998
	T2 Wk0 vs Wk3 (Dirty)	4.706	0.370	**0.017**	0.066
	T2 Wk1 vs Wk2	0.998	0.111	0.524	0.999
	T2 Wk1 vs Wk3 (Clean)	3.335	0.294	**0.016**	0.997
	T2 Wk1 vs Wk3 (Dirty)	4.417	0.356	**0.014**	0.057
	T2 Wk2 vs Wk3 (Clean)	3.673	0.315	**0.015**	0.866
	T2 Wk2 vs Wk3 (Dirty)	4.765	0.373	**0.019**	**0.009**
	T2 Wk3 (Clean) vs Wk3 (Dirty)	4.515	0.361	**0.015**	0.288
