# Supplementary material for: Eggshell and environmental bacteria contribute to the intestinal microbiota of growing chickens
Source: J Anim Sci Biotechnol. 2020 Jun 11;11:60. doi: 10.1186/s40104-020-00459-w (PMC7288515; doi:10.1186/s40104-020-00459-w)
Supplement: Supplementary file 4 — Additional file 4: Table S4. Significant alpha diversity comparison results for the lumen- and mucosa-associated microbial communities in the cecum. Comparisons are labeled input group_week_trial-input group_week_trial. Comparisons between microbiota input groups, trials, and weeks were made using an analysis of variance (ANOVA) followed by subsequent pairwise comparisons with the Tukey’s “Honest Significant Difference” method. Only statistically significant comparisons are included in the table (P < 0.05). [file 40104_2020_459_MOESM4_ESM.pdf]

Cecal Lumen

T1 only	p.adj	T2 only	p.adj	Between Trials	p.adj
CONV_Wk3_T1-CONV_Wk1_T1	0.0015	CONV_Wk3_T2-CONV_Wk1_T2	0.0009	CONV_Wk1_T2-CONV_Wk1_T1	0.0001
CONV_Wk6_T1-CONV_Wk1_T1	0.0000	CONV_Wk6_T2-CONV_Wk1_T2	0.0000	CONV_Wk3_T2-CONV_Wk3_T1	0.0004
EGG_Wk1_T1-CONV_Wk1_T1	0.0000	EGG_Wk1_T2-CONV_Wk1_T2	0.0000	EGG_Wk3_T2-EGG_Wk3_T1	0.0000
ENV_Wk1_T1-CONV_Wk1_T1	0.0000	ENV_Wk1_T2-CONV_Wk1_T2	0.0014	EGG_Wk6_T2-EGG_Wk6_T1	0.0000
CONV_Wk6_T1-CONV_Wk3_T1	0.0000	EGG_Wk3_T2-CONV_Wk3_T2	0.0000	ENV_Wk1_T2-ENV_Wk1_T1	0.0006
EGG_Wk3_T1-CONV_Wk3_T1	0.0002	ENV_Wk3_T2-CONV_Wk3_T2	0.0000	ENV_Wk3_T2-ENV_Wk3_T1	0.0380
ENV_Wk3_T1-CONV_Wk3_T1	0.0000	EGG_Wk6_T2-CONV_Wk6_T2	0.0000		
ENV_Wk6_T1-CONV_Wk6_T1	0.0000	ENV_Wk6_T2-CONV_Wk6_T2	0.0000		
ENV_Wk1_T1-EGG_Wk1_T1	0.0000	EGG_Wk3_T2-EGG_Wk1_T2	0.0000		
ENV_Wk3_T1-EGG_Wk3_T1	0.0000	EGG_Wk6_T2-EGG_Wk1_T2	0.0000		
ENV_Wk6_T1-EGG_Wk6_T1	0.0000	ENV_Wk3_T2-EGG_Wk3_T2	0.0035		
ENV_Wk3_T1-ENV_Wk1_T1	0.0000	ENV_Wk3_T2-ENV_Wk1_T2	0.0000		
ENV_Wk6_T1-ENV_Wk1_T1	0.0000	ENV_Wk6_T2-ENV_Wk1_T2	0.0000		

Cecal Mucosa

T1 only	p.adj	T2 only	p.adj	Between Trials	p.adj
CONV_Wk6_T1-CONV_Wk1_T1	0.0000	CONV_Wk3_T2-CONV_Wk1_T2	0.0030	CONV_Wk1_T2-CONV_Wk1_T1	0.0262
EGG_Wk1_T1-CONV_Wk1_T1	0.0010	CONV_Wk6_T2-CONV_Wk1_T2	0.0000	CONV_Wk3_T2-CONV_Wk3_T1	0.0002
ENV_Wk1_T1-CONV_Wk1_T1	0.0000	EGG_Wk1_T2-CONV_Wk1_T2	0.0049	EGG_Wk3_T2-EGG_Wk3_T1	0.0000
CONV_Wk6_T1-CONV_Wk3_T1	0.0000	CONV_Wk6_T2-CONV_Wk3_T2	0.0051	EGG_Wk6_T2-EGG_Wk6_T1	0.0011
ENV_Wk3_T1-CONV_Wk3_T1	0.0000	EGG_Wk3_T2-CONV_Wk3_T2	0.0000		
EGG_Wk6_T1-CONV_Wk6_T1	0.0262	ENV_Wk3_T2-CONV_Wk3_T2	0.0000		
ENV_Wk6_T1-CONV_Wk6_T1	0.0000	EGG_Wk3_T2-EGG_Wk1_T2	0.0000		
ENV_Wk3_T1-EGG_Wk3_T1	0.0000	EGG_Wk6_T2-EGG_Wk1_T2	0.0001		
ENV_Wk6_T1-EGG_Wk6_T1	0.0000	ENV_Wk3_T2-ENV_Wk1_T2	0.0000		
ENV_Wk3_T1-ENV_Wk1_T1	0.0000	ENV_Wk6_T2-ENV_Wk1_T2	0.0000		
ENV_Wk6_T1-ENV_Wk1_T1	0.0000				
