# Supplementary material for: Eggshell and environmental bacteria contribute to the intestinal microbiota of growing chickens
Source: J Anim Sci Biotechnol. 2020 Jun 11;11:60. doi: 10.1186/s40104-020-00459-w (PMC7288515; doi:10.1186/s40104-020-00459-w)
Supplement: Supplementary file 5 — Additional file 5: Table S5. Comparisons of beta diversity (PERMANOVA) for the lumen- and mucosa-associated microbial communities at weeks 1, 3, and 6 for T1 and T2 birds. Only statistically significant comparisons are included in the Table (P < 0.05). Bold values represent statistical significance as determined by PERMANOVA or beta dispersion (P < 0.05). [file 40104_2020_459_MOESM5_ESM.pdf]

T1			F.Model	R2	p.fdr	T2			F.Model	R2	p.fdr
CONV	Cecum	Wk1	1.75	0.10	0.171	CONV	Cecum	Wk1	1.83	0.08	0.145
		Wk3	5.43	0.23	**0.002**			Wk3	5.27	0.20	**0.001**
		Wk6	6.76	0.25	**0.002**			Wk6	7.68	0.27	**0.001**
	Ileum	Wk1	1.24	0.07	0.326		Ileum	Wk1	0.44	0.03	0.8
		Wk3	1.69	0.11	0.187			Wk3	25.41	0.55	**0.001**
		Wk6	12.23	0.42	**0.002**			Wk6	11.25	0.34	**0.002**
	Jejunum	Wk1	1.75	0.11	0.183		Jejunum	Wk1	4.76	0.22	**0.006**
		Wk3	0.89	0.06	0.442			Wk3	8.87	0.37	**0.001**
		Wk6	4.47	0.20	**0.022**			Wk6	0.78	0.05	0.457
EGG	Cecum	Wk1	1.84	0.19	0.172	EGG	Cecum	Wk1	3.20	0.10	**0.005**
		Wk3	1.32	0.14	0.221			Wk3	11.45	0.34	**0.001**
		Wk6	2.86	0.26	**0.011**			Wk6	14.67	0.41	**0.001**
	Ileum	Wk1	4.39	0.35	**0.018**		Ileum	Wk1	1.19	0.05	0.33
		Wk3	4.38	0.35	**0.025**			Wk3	2.77	0.11	**0.03**
		Wk6	2.16	0.21	0.055			Wk6	7.05	0.24	**0.002**
	Jejunum	Wk1	7.17	0.47	**0.009**		Jejunum	Wk1	2.49	0.10	0.079
		Wk3	1.09	0.12	0.289			Wk3	3.62	0.17	**0.009**
		Wk6	1.18	0.13	0.315			Wk6	6.87	0.24	**0.001**
ENV	Cecum	Wk1	0.77	0.03	0.619	ENV	Cecum	Wk1	2.27	0.09	**0.043**
		Wk3	2.87	0.12	**0.012**			Wk3	5.83	0.23	**0.001**
		Wk6	3.26	0.13	**0.002**			Wk6	2.21	0.09	**0.007**
	Ileum	Wk1	2.22	0.10	0.054		Ileum	Wk1	9.70	0.31	**0.001**
		Wk3	2.60	0.11	**0.009**			Wk3	4.03	0.17	**0.005**
		Wk6	2.78	0.11	**0.045**			Wk6	3.35	0.16	**0.021**
	Jejunum	Wk1	1.98	0.12	0.055		Jejunum	Wk1	4.39	0.24	**0.002**
		Wk3	2.67	0.23	**0.038**			Wk3	3.55	0.21	**0.002**
		Wk6	1.35	0.07	0.238			Wk6	3.68	0.20	**0.018**
