# Supplementary material for: Eggshell and environmental bacteria contribute to the intestinal microbiota of growing chickens
Source: J Anim Sci Biotechnol. 2020 Jun 11;11:60. doi: 10.1186/s40104-020-00459-w (PMC7288515; doi:10.1186/s40104-020-00459-w)
Supplement: Supplementary file 6 — Additional file 6: Figure S1. Stacked barcharts comparing the weekly relative abundance of bacterial phyla (> 0.1%) and genera (> 2.0%) of the eggshell bacterial community at any time point within T1 and T2. [file 40104_2020_459_MOESM6_ESM.pdf]

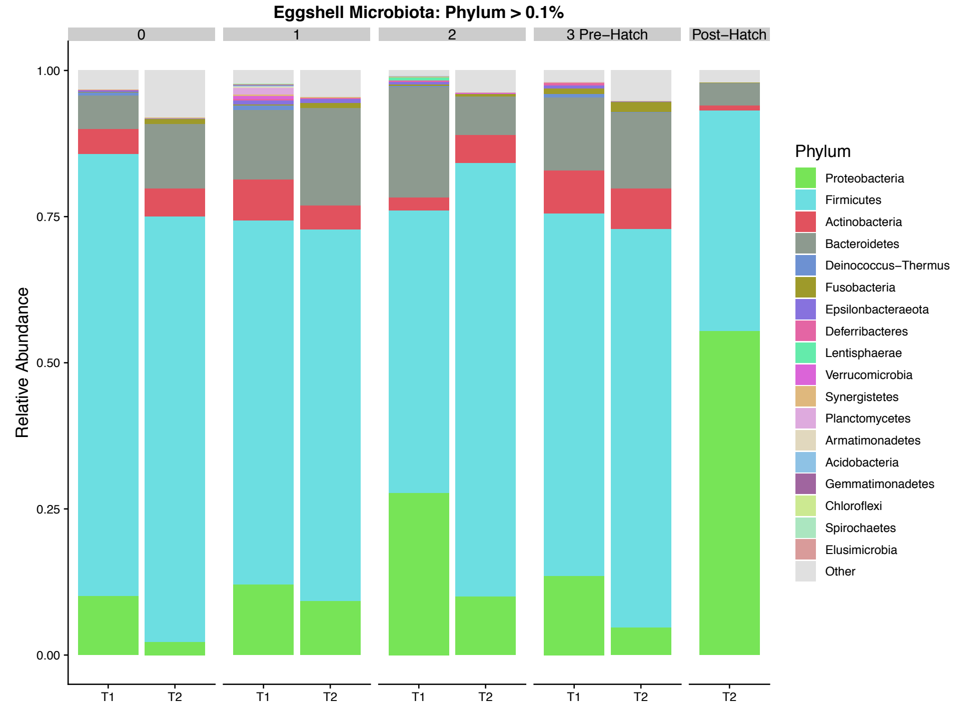
Eggshell Microbiota: Phylum > 0.1%
0
1
2
3 Pre–Hatch
Post–Hatch
Relative Abundance
1.00
0.75
0.50
0.25
0.00
T1
T2
Phylum
Proteobacteria
Firmicutes
Actinobacteria
Bacteroidetes
Deinococcus–Thermus
Fusobacteria
Epsilonbacteraeota
Deferribacteres
Lentisphaerae
Verrucomicrobia
Synergistetes
Planctomycetes
Armatimonadetes
Acidobacteria
Gemmatimonadetes
Chloroflexi
Spirochaetes
Elusimicrobia
Other

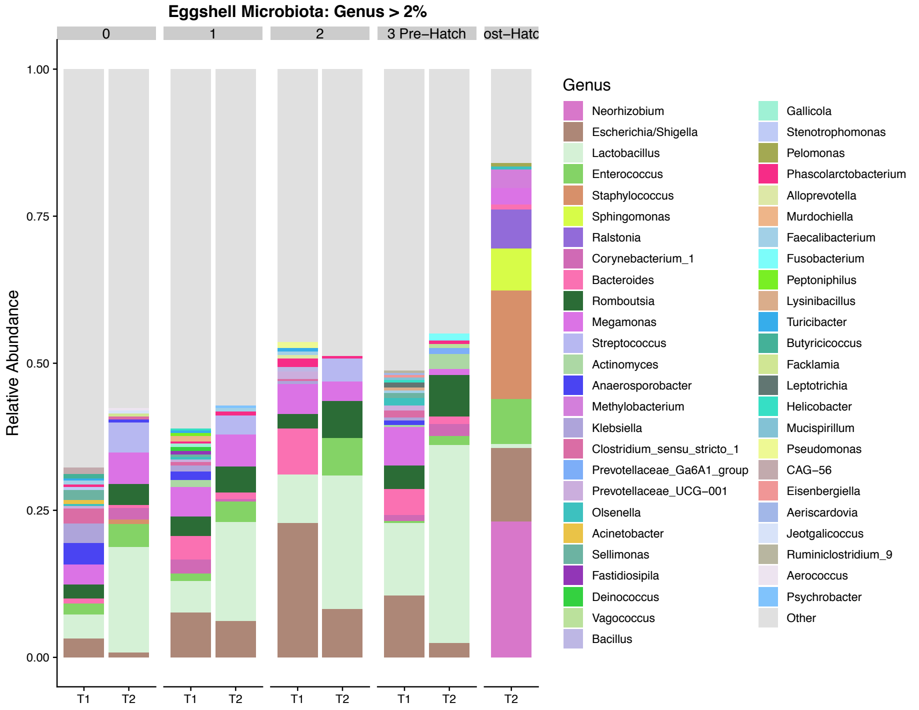
Eggshell Microbiota: Genus > 2%
0
1
2
3 Pre–Hatch
ost–Hatc
Relative Abundance
1.00
0.75
0.50
0.25
0.00
T1
T2
Genus
Neorhizobium
Escherichia/Shigella
Lactobacillus
Enterococcus
Staphylococcus
Sphingomonas
Ralstonia
Corynebacterium_1
Bacteroides
Romboutsia
Megamonas
Streptococcus
Actinomyces
Anaerosporobacter
Methylobacterium
Klebsiella
Clostridium_sensu_stricto_1
Prevotellaceae_Ga6A1_group
Prevotellaceae_UCG–001
Olsenella
Acinetobacter
Sellimonas
Fastidiosipila
Deinococcus
Vagococcus
Bacillus
Gallicola
Stenotrophomonas
Pelomonas
Phascolarctobacterium
Alloprevotella
Murdochiella
Faecalibacterium
Fusobacterium
Peptoniphilus
Lysinibacillus
Turicibacter
Butyricicoccus
Facklamia
Leptotrichia
Helicobacter
Mucispirillum
Pseudomonas
CAG–56
Eisenbergiella
Aeriscardovia
Jeotgalicoccus
Ruminiclostridium_9
Aerococcus
Psychrobacter
Other
