# Supplementary figures and images for: Eggshell and environmental bacteria contribute to the intestinal microbiota of growing chickens
Source: J Anim Sci Biotechnol. 2020 Jun 11;11:60. doi: 10.1186/s40104-020-00459-w (PMC7288515; doi:10.1186/s40104-020-00459-w)

Fecal Swab Microbiota: Phylum &gt; 1%

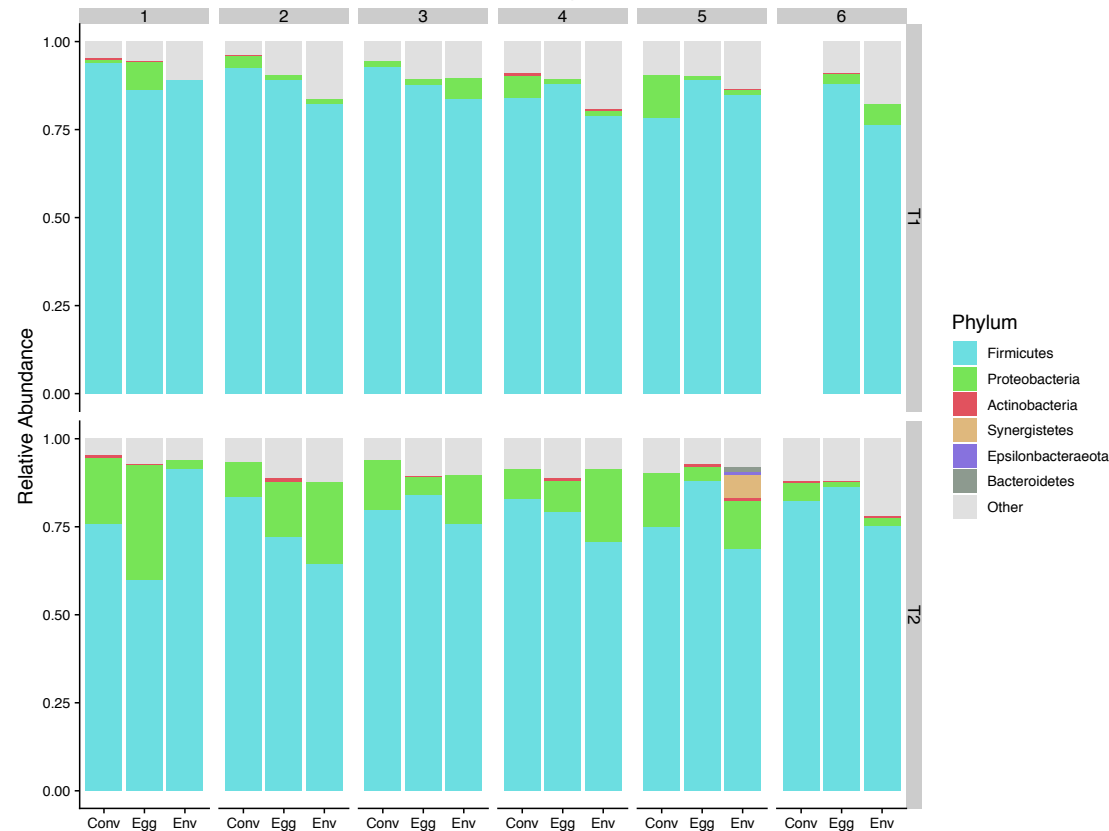

Fecal Swab Microbiota: Genus &gt; 2%

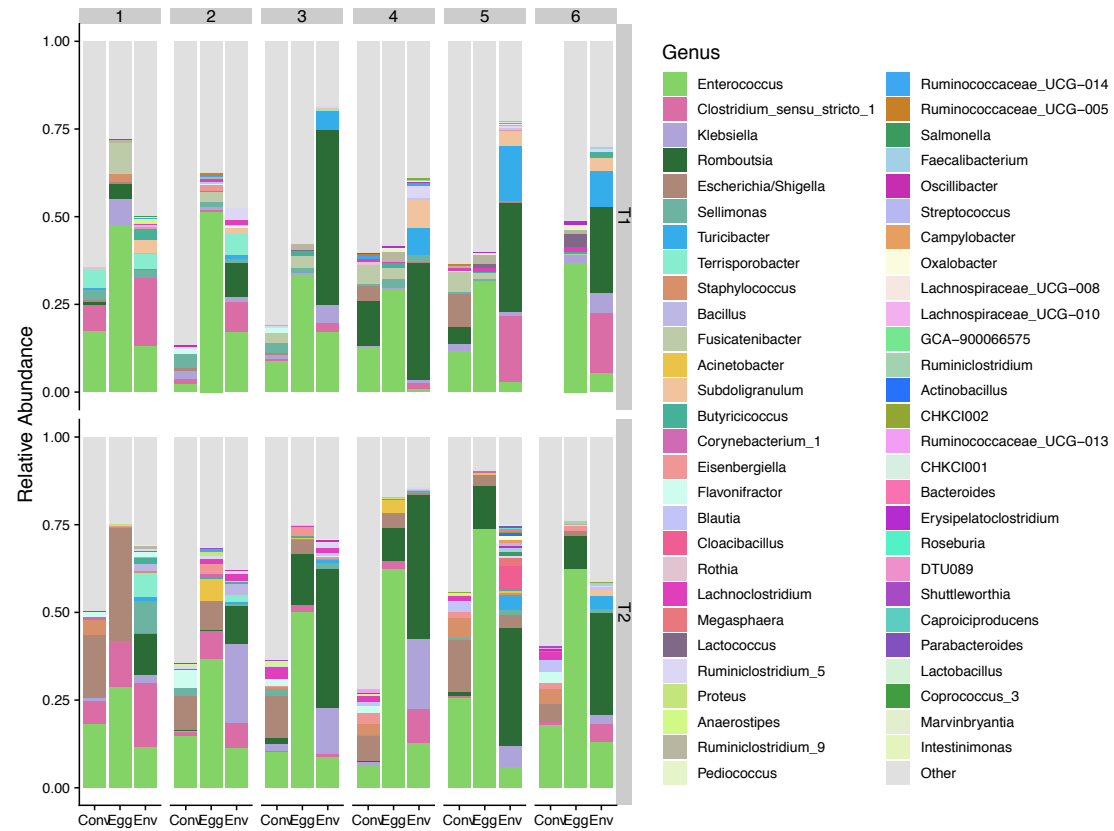

Supplement: Supplementary file 7 — Additional file 7: Figure S2. Stacked barcharts comparing the weekly relative abundance of bacterial phyla (> 1.0%) and genera (> 2.0%) of the fecal swab bacterial community for T1 and T2. [file 40104_2020_459_MOESM7_ESM.pdf]

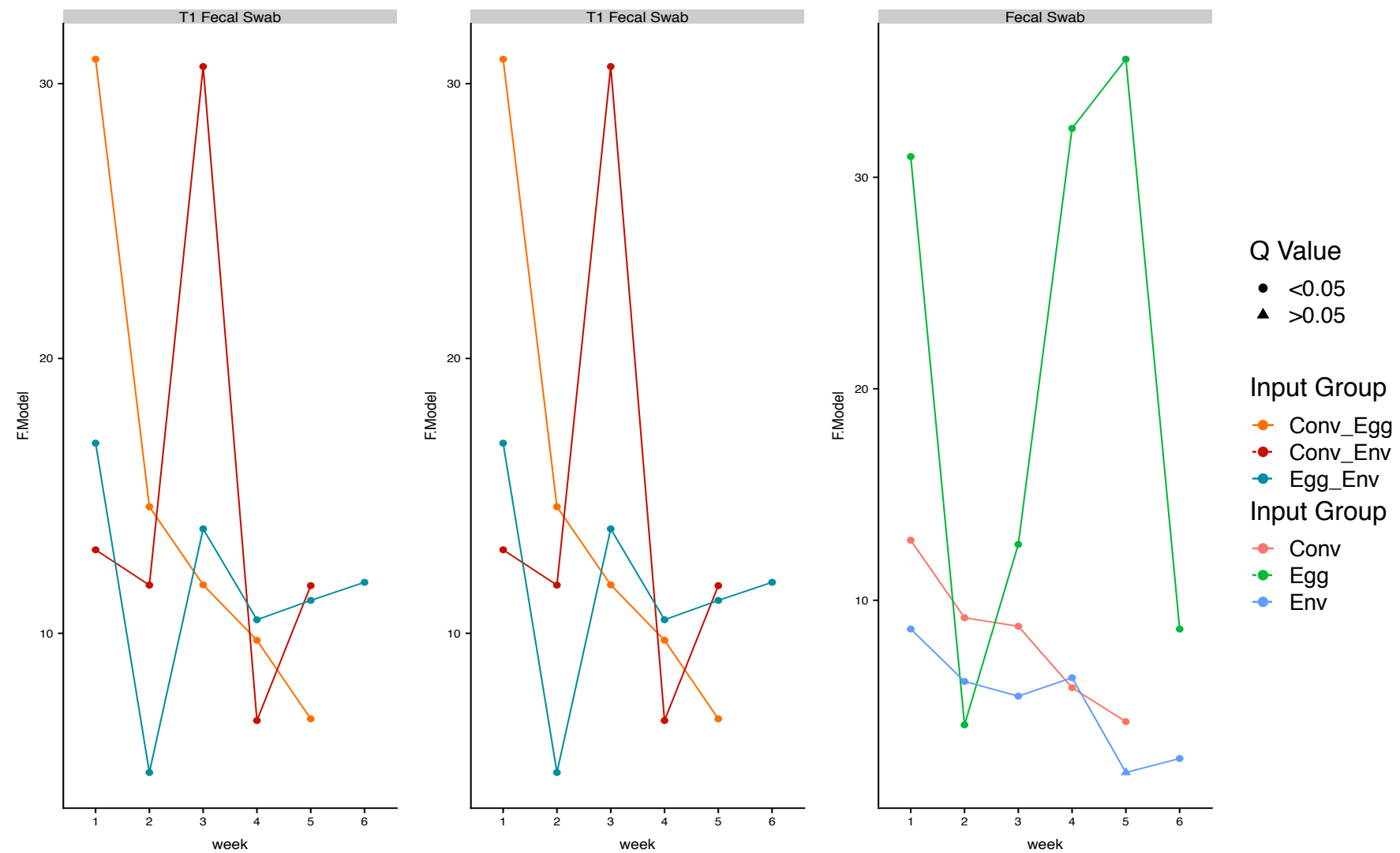

Supplement: Supplementary file 8 — Additional file 8: Figure S3. Weekly population level PERMANOVA statistics (F.models and q-values) for the fecal swabs between microbiota input groups within the same trial and between trials within the same input group. [file 40104_2020_459_MOESM8_ESM.pdf]

**A****T1 Relative Abundance: Phylum > 1%**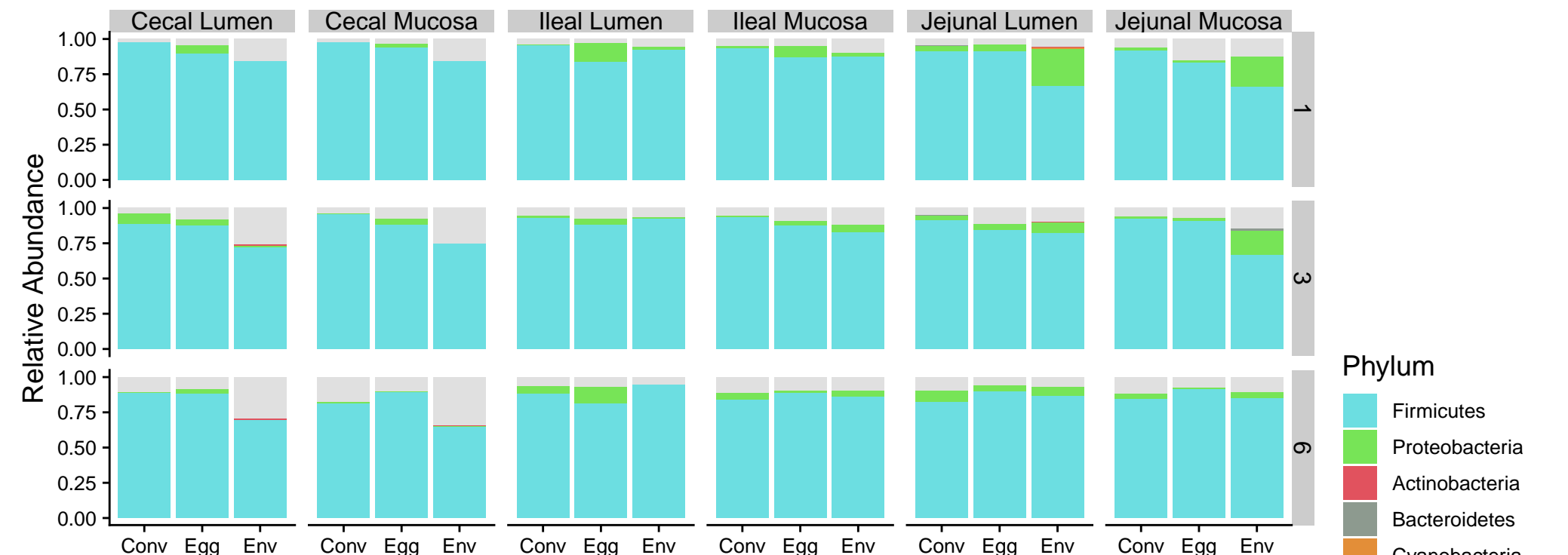**B****T2 Relative Abundance: Phylum > 1%**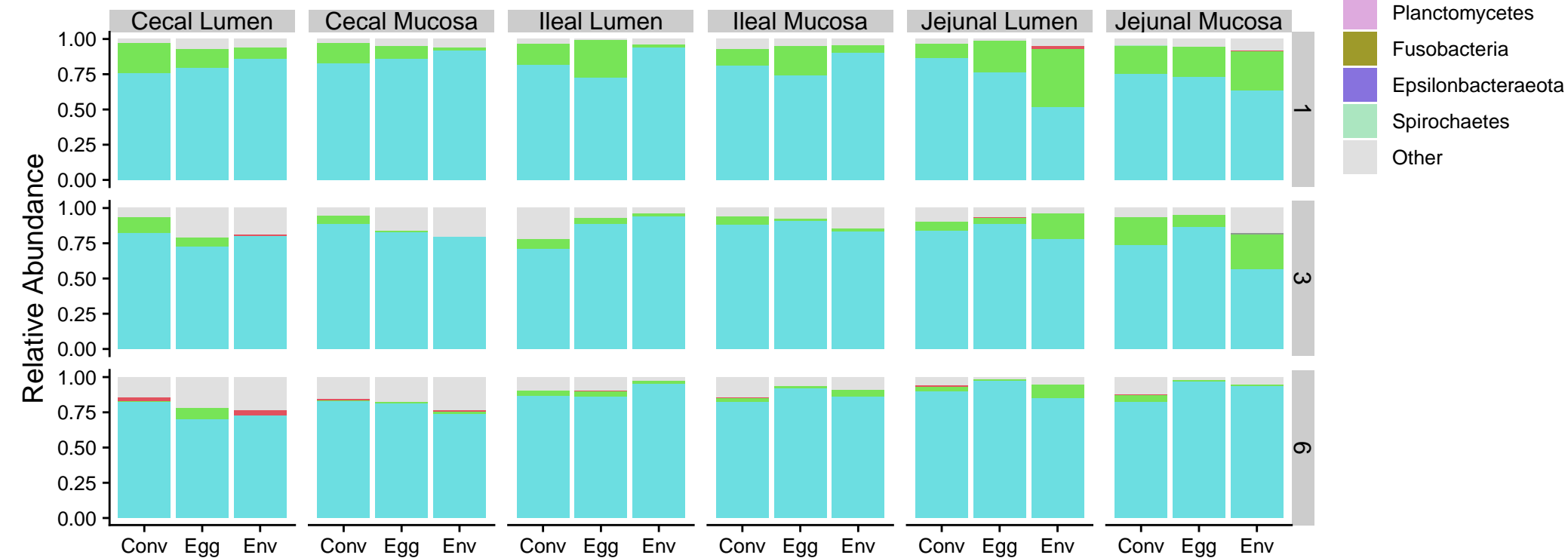

Supplement: Supplementary file 9 — Additional file 9: Figure S4. Stacked barcharts comparing the relative abundance of bacterial phyla (> 1.0%) along the intestinal tract, both lumen and mucosa, at weeks 1, 3, and 6 for T1 and T2 birds. [file 40104_2020_459_MOESM9_ESM.pdf]

**T1 Relative Abundance: Genus > 2%**

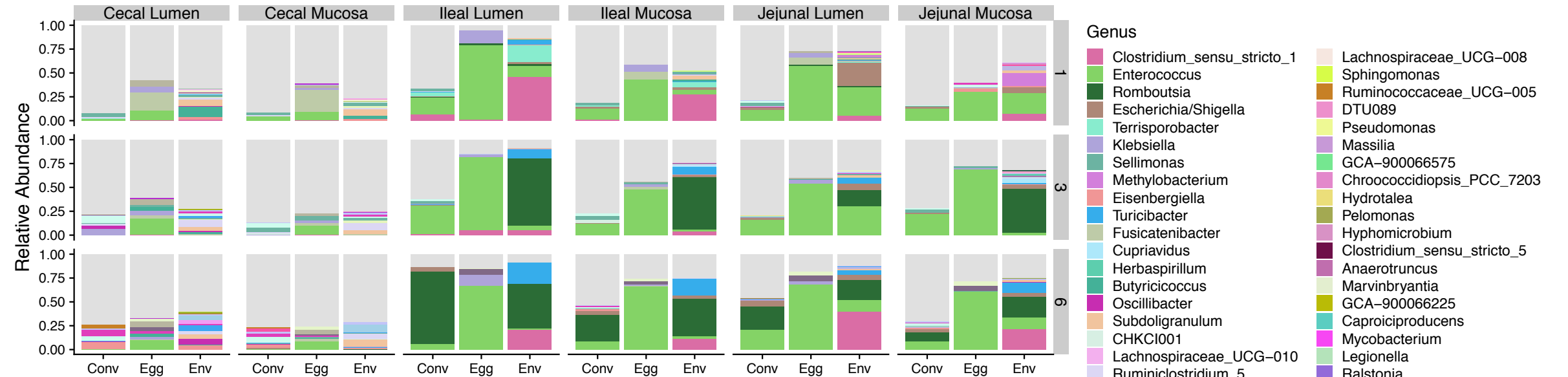

**T2 Relative Abundance: Genus > 2%**

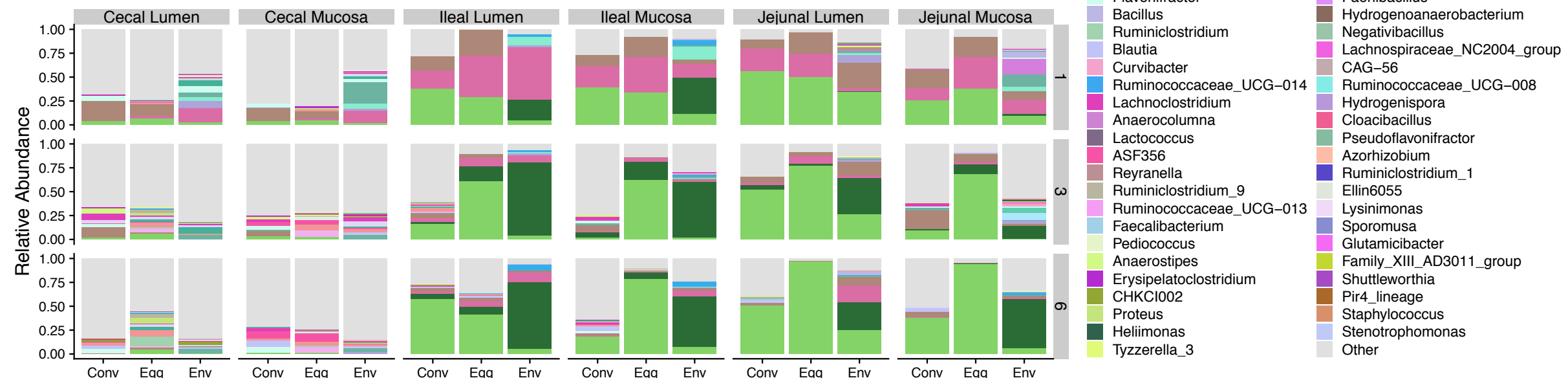

Supplement: Supplementary file 10 — Additional file 10: Figure S5. Stacked barcharts comparing the relative abundance of bacterial genera (> 2.0%) along the intestinal tract, both lumen and mucosa, at weeks 1, 3, and 6 for T1 and T2 birds. [file 40104_2020_459_MOESM10_ESM.pdf]

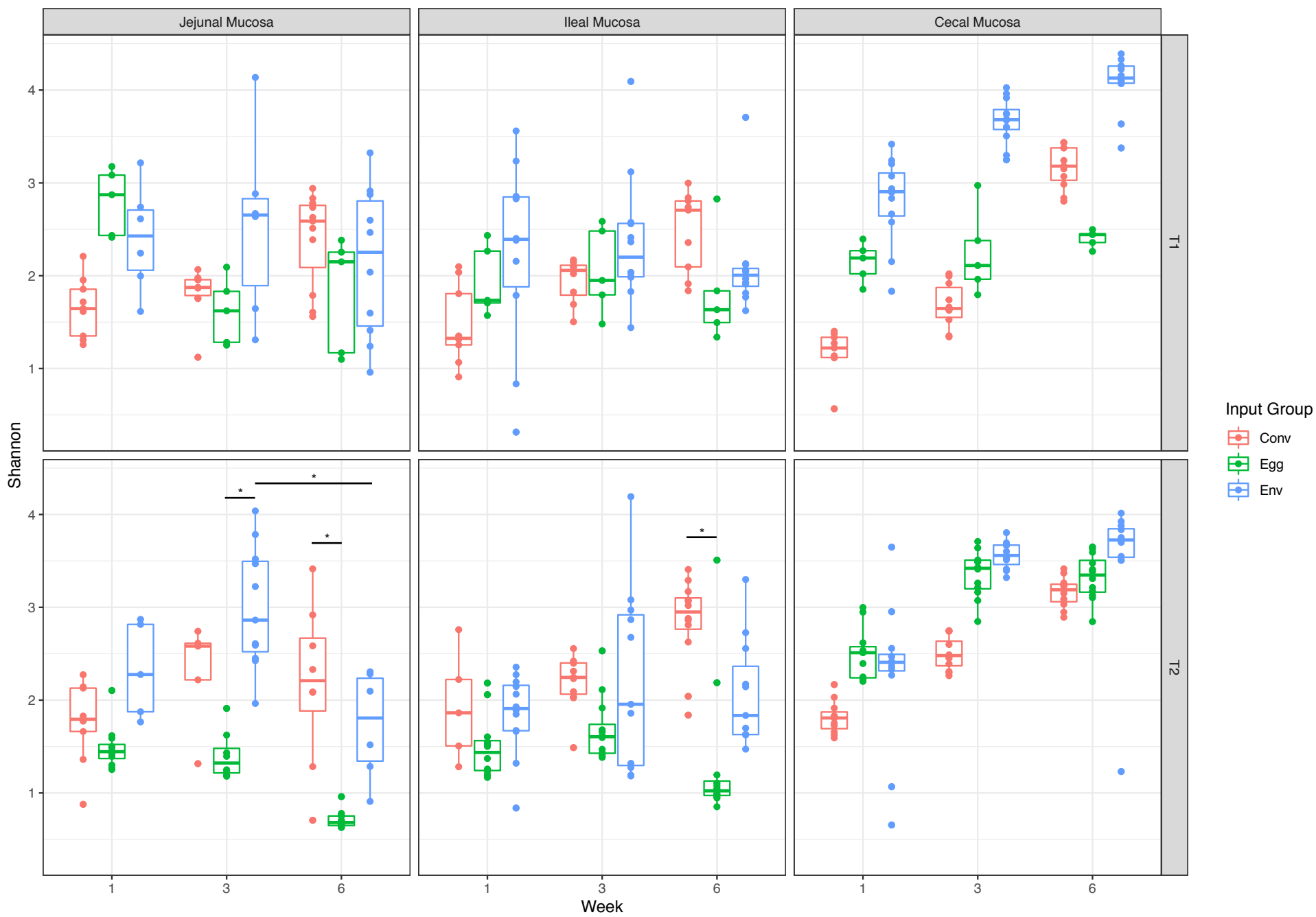

Supplement: Supplementary file 15 — Additional file 15: Figure S10. Changes in mucosal microbiota alpha-diversity (Shannon index) for different microbiota input groups throughout the 6-week trial period for both T1 and T2. Comparisons between Shannon indices between microbiota input groups, weeks, and trials were made using an analysis of variance (ANOVA) followed by subsequent pairwise comparisons with the Tukey’s “Honest Significant Difference” method. Significance bars with an asterisk (*) indicate difference between microbiota input groups and/or time points (P < 0.05). [file 40104_2020_459_MOESM15_ESM.pdf]

**T1 Relative Abundance: Family > 1%**

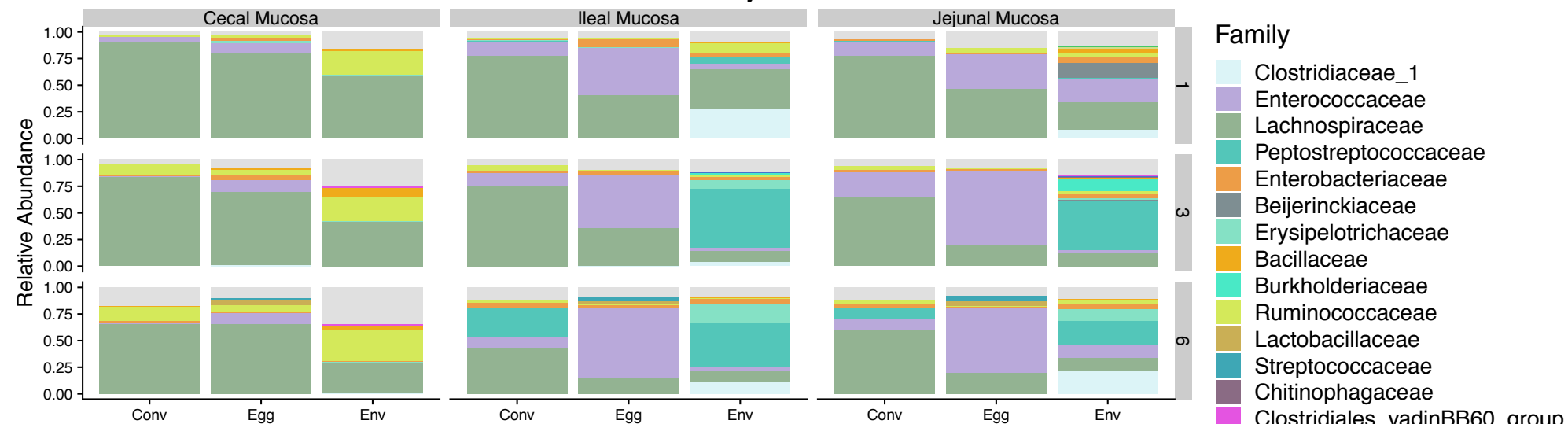

**T2 Relative Abundance: Family > 1%**

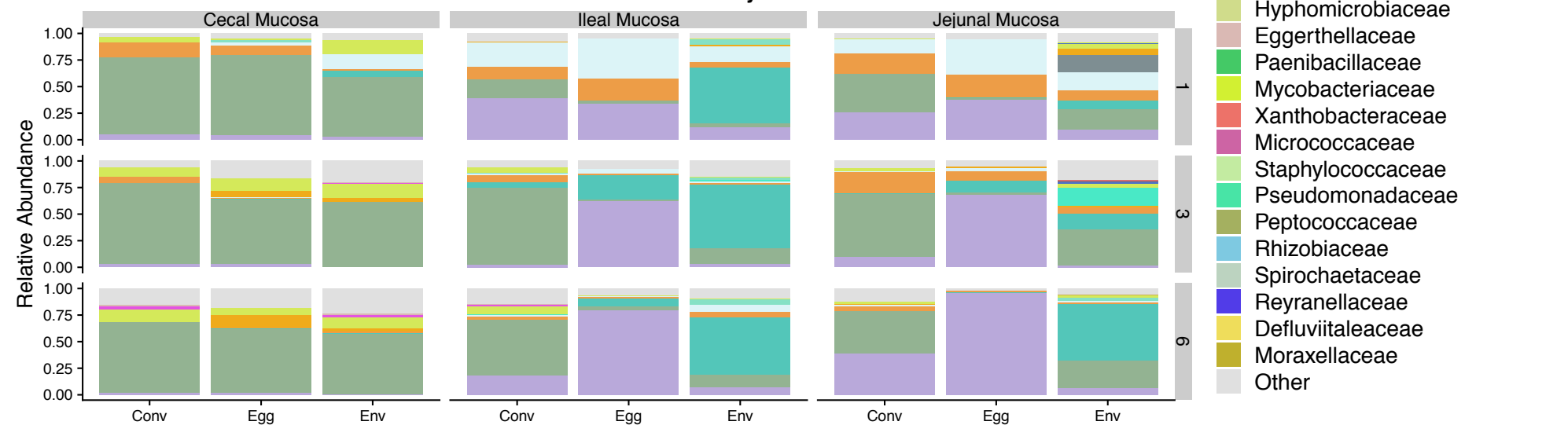

Supplement: Supplementary file 16 — Additional file 16: Figure S11. Stacked barcharts comparing the relative abundance of bacterial families (> 1.0%) along the intestinal mucosa at weeks 1, 3, and 6 for T1 and T2 birds. [file 40104_2020_459_MOESM16_ESM.pdf]

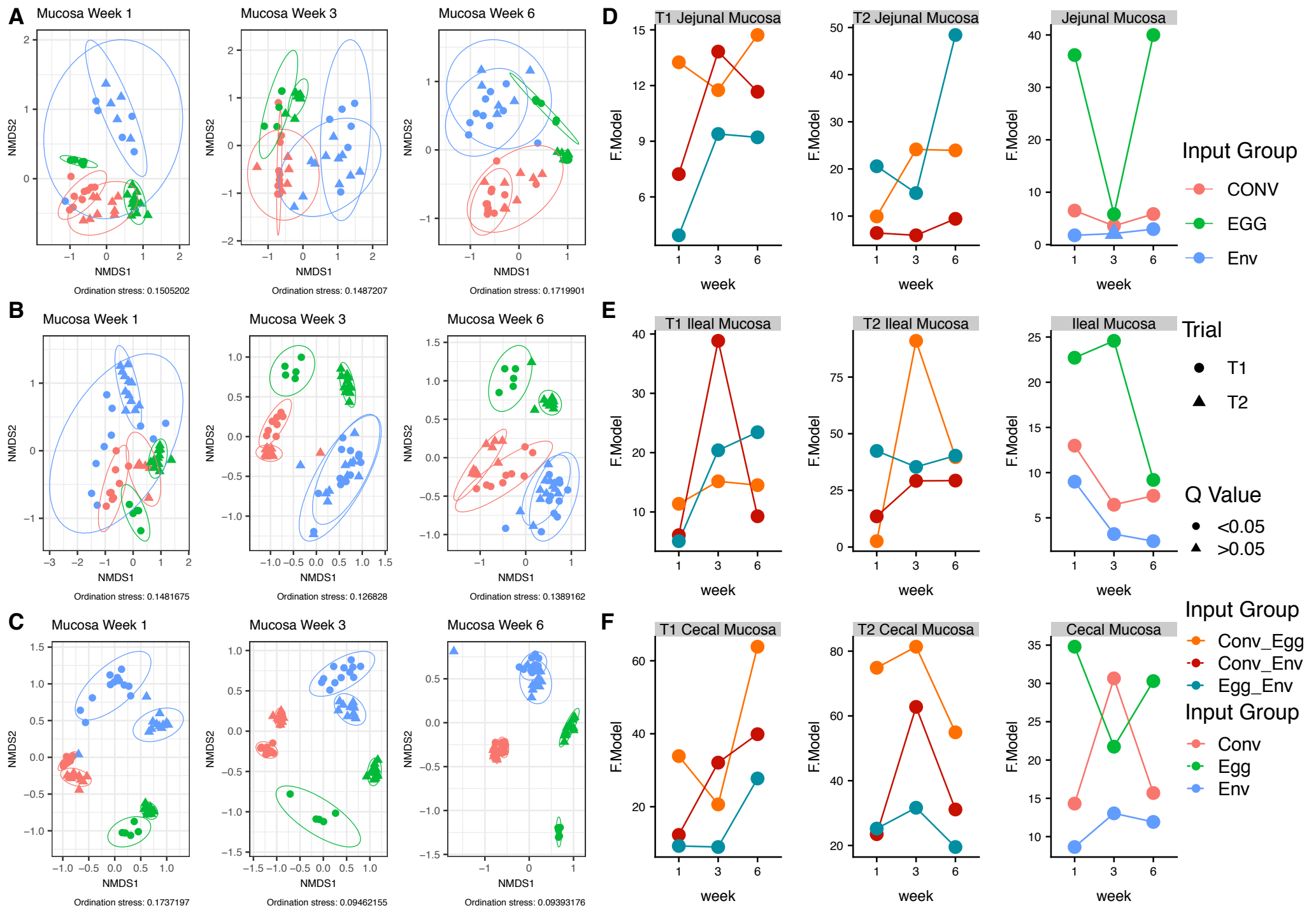

Supplement: Supplementary file 17 — Additional file 17: Figure S12. Beta-diversity of mucosal bacterial communities between different microbiota input groups within the (A) jejunum, (B) ileum, and (C) cecum for both T1 and T2 at weeks 1, 3, and 6. Ellipses were generated around points to aid in visualizing group differences assuming a multivariate T-distribution with a 95% confidence interval. Population level PERMANOVA statistics (F.models and q-values) were also assessed between microbiota input groups within the same trial and between trials within the same input group for the (D) jejunum, (E) ileum, and (F) cecum. [file 40104_2020_459_MOESM17_ESM.pdf]
