# Supplementary material for: Eggshell and environmental bacteria contribute to the intestinal microbiota of growing chickens
Source: J Anim Sci Biotechnol. 2020 Jun 11;11:60. doi: 10.1186/s40104-020-00459-w (PMC7288515; doi:10.1186/s40104-020-00459-w)

Highly abundant ASVs (>1.0% in at least one input group) shared between T1 and T2 bird Ceca

T1 bird cecal ASV abundances: Conv_Abund	Egg_Abund	Env_Abund	ASV and Taxonomy	T2 bird cecal ASV abundances: Conv_Abund	Egg_Abund	Env_Abund
**1.78**	**8.638**	0.162	ASV_1_Enterococcaceae_Enterococcus	**1.4**	**4.761**	**1.597**
**37.089**	**2.013**	**4.099**	ASV_3_Lachnospiraceae_NA	**21.056**	0.027	**4.113**
**16.655**	**24.437**	**3.231**	ASV_4_Lachnospiraceae_NA	**18.404**	0.582	0.416
**9.025**	–	**1.193**	ASV_7_Lachnospiraceae_NA	**9.209**	0.217	0.003
**1.506**	**4.929**	**1.807**	ASV_8_Lachnospiraceae_NA	0.118	**12.176**	**3.273**
**1.412**	**3.683**	0.343	ASV_10_Enterobacteriaceae_Klebsiella	0.42	0.004	**2.092**
0.134	**1.951**	0.097	ASV_12_Enterococcaceae_Enterococcus	**2.046**	0.391	0.006
**4.018**	0.014	0.962	ASV_16_Ruminococcaceae_Flavonifractor	**4.575**	0.004	**2.134**
0.036	**1.398**	**3.867**	ASV_19_Lachnospiraceae_NA	0.054	0.471	**5.305**
0.607	–	**2.048**	ASV_20_Lachnospiraceae_Eisenbergiella	0.343	**4.202**	**1.523**
**1.916**	–	**2.218**	ASV_21_Lachnospiraceae_NA	**1.577**	–	0.106
**1.247**	–	0.876	ASV_22_Lachnospiraceae_Lachnoclostridium	**2.243**	0	0.782
0.096	0.99	**2.793**	ASV_24_Bacillaceae_NA	0.001	**3.757**	**1.287**
**1.358**	–	**1.28**	ASV_27_Lachnospiraceae_Blautia	**1.528**	–	0.972
**2.436**	–	0.295	ASV_30_Lachnospiraceae_NA	**2.552**	–	0.001
0.047	0.564	**2.511**	ASV_33_Lachnospiraceae_NA	0.04	**2.607**	**1.03**
0.022	0	**1.763**	ASV_47_Lachnospiraceae_NA	0.003	0.817	**2.046**
**1.495**	–	0.426	ASV_48_Ruminococcaceae_Ruminiclostridium_5	**1.035**	0	0.947
**1.35**	0.056	0.081	ASV_49_Ruminococcaceae_Oscillibacter	**1.753**	0.001	0.489

Highly abundant ASVs (>1.0% in at least one input group) exclusive to either T1 or T2 bird ceca

.

ASVs exclusive to T1 bird ceca and abundances

Conv_Abund	Egg_Abund	Env_Abund	ASV and Taxonomy
**1.083**	–	0.258	ASV_75_Ruminococcaceae_Ruminococcaceae_UCG-005
**1.271**	–	0.146	ASV_87_Ruminococcaceae_NA
0.007	–	**2.018**	ASV_95_Ruminococcaceae_Butyricicoccus

ASVs exclusive to T2 bird ceca and abundances

ASV and Taxonomy	Conv_Abund	Egg_Abund	Env_Abund
ASV_5_Clostridiaceae_1_Clostridium_sensu_stricto_1	0.154	**1.659**	0.41
ASV_6_Enterobacteriaceae_Escherichia/Shigella	**8.8**	**4.833**	0.039
ASV_11_Clostridiaceae_1_Clostridium_sensu_stricto_1	0.029	0.084	**2.575**
ASV_14_Lachnospiraceae_NA	0.005	**3.272**	0.126
ASV_15_Lachnospiraceae_NA	0.041	**4.864**	**7.859**
ASV_25_Ruminococcaceae_Ruminiclostridium_9	0	**1.605**	**1.114**
ASV_28_Lachnospiraceae_NA	0.457	**4.623**	**1.483**
ASV_29_Lachnospiraceae_NA	0.004	0.969	**8.045**
ASV_34_Ruminococcaceae_Ruminiclostridium_5	0.012	**1.185**	**1.857**
ASV_36_Lachnospiraceae_NA	0.026	**3.017**	0.955
ASV_38_Lachnospiraceae_Sellimonas	0.026	0.07	**5.446**
ASV_43_Ruminococcaceae_Butyricicoccus	0.331	**1.447**	**2.206**
ASV_46_Lachnospiraceae_Eisenbergiella	**1.073**	–	0.354
ASV_53_Lachnospiraceae_ASF356	**1.605**	**1.89**	0.601
ASV_58_Clostridiales_vadinBB60_group_NA	**1.597**	0.001	**1.482**
ASV_64_Lachnospiraceae_NA	0.153	**2.395**	–
ASV_66_Lachnospiraceae_Anaerostipes	**1.437**	0	–
ASV_69_Enterobacteriaceae_Proteus	0.009	**2.475**	–
ASV_78_Lachnospiraceae_ASF356	**1.05**	0.943	0.246
ASV_80_Eggerthellaceae_CHKCI002	0.728	0	**1.264**
ASV_97_Ruminococcaceae_Ruminiclostridium	0.001	**2.162**	–
ASV_104_Clostridiaceae_1_Clostridium_sensu_stricto_1	0.009	0.065	**1.399**

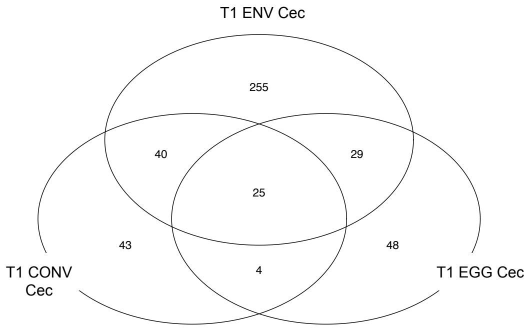

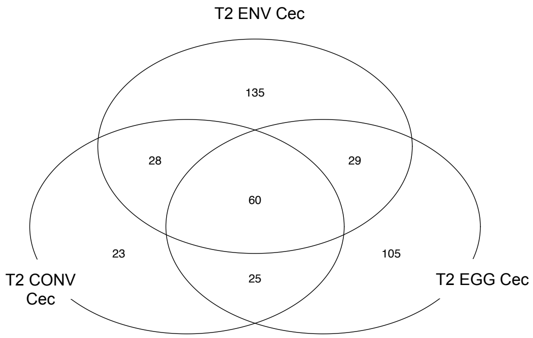

Supplement: Supplementary file 11 — Additional file 11: Figure S6. List of cecum-associated bacterial ASVs shared between bacterial input groups and trials. ASVs are listed with a number followed by the associated family and genus classifications and a number if there are multiple, unique ASVs with the same family and genus classifications. Bolded numbers are > 1.0% relative abundance. Core shared ASVs in the upper table are those present at > 1.0% relative abundance in a least one of the microbial input groups in both trials. ASVs present at > 1.0% relative abundance in a least one of the microbial input groups in only one trial are found in the lower potion of the table. Venn diagrams at the bottom of the figure show the sharing of ASVs between microbial input groups with > 10 reads within a trial. [file 40104_2020_459_MOESM11_ESM.pdf]
