# Supplementary material for: Eggshell and environmental bacteria contribute to the intestinal microbiota of growing chickens
Source: J Anim Sci Biotechnol. 2020 Jun 11;11:60. doi: 10.1186/s40104-020-00459-w (PMC7288515; doi:10.1186/s40104-020-00459-w)

Highly abundant ASVs (>1.0% in at least one input group) shared between T1 and T2 bird ileum

T1 bird ileal ASV abundances: Conv_Abund	Egg_Abund	Env_Abund	ASV and Taxonomy	T2 bird ileal ASV abundances: Conv_Abund	Egg_Abund	Env_Abund
**12.125**	**53.63**	**4.849**	ASV_1_Enterococcaceae_Enterococcus	**24.944**	**45.751**	**6.348**
**15.779**	0.274	**30.389**	ASV_2_Peptostreptococcaceae_Romboutsia	**2.734**	**8.455**	**45.507**
**29.749**	**5.145**	**2.743**	ASV_3_Lachnospiraceae_NA	**11.042**	0.881	**1.357**
**9.639**	**5.332**	**1.47**	ASV_4_Lachnospiraceae_NA	**7.553**	**1.621**	0.258
0.013	0.028	**14.117**	ASV_5_Clostridiaceae_1_Clostridium_sensu_stricto_1	**6.141**	**15.197**	**2.396**
**2.545**	0.274	**2.01**	ASV_6_Enterobacteriaceae_Escherichia/Shigella	**6.713**	**9.808**	**2.064**
**5.711**	0.027	0.364	ASV_7_Lachnospiraceae_NA	**3.209**	0.188	0.099
0.419	**6.804**	0.478	ASV_10_Enterobacteriaceae_Klebsiella	0.097	0.018	**1.196**
**1.71**	**1.441**	**4.577**	ASV_11_Clostridiaceae_1_Clostridium_sensu_stricto_1	0.777	0.306	**13.155**
0.166	**2.207**	0.036	ASV_12_Enterococcaceae_Enterococcus	**1.323**	0.242	0.021
0.174	0.002	**11.128**	ASV_13_Erysipelotrichaceae_Turicibacter	0	0.003	**4.116**
**1.112**	0.217	0.131	ASV_16_Ruminococcaceae_Flavonifractor	**1.129**	0.101	0.158
**2.029**	0.024	**2.305**	ASV_23_Peptostreptococcaceae_Romboutsia	0.067	0.369	**1.996**
**1.033**	0.007	**3.684**	ASV_26_Peptostreptococcaceae_Terrisporobacter	0	–	**4.538**
0.326	–	**1.51**	ASV_45_Peptostreptococcaceae_Romboutsia	0.029	0.422	**2.482**

Highly abundant ASVs (>1.0% in at least one input group) exclusive to either T1 or T2 bird ileum

Conv_Abund	Egg_Abund	Env_Abund	ASVs exclusive to T1 bird ileum and abundances
0.004	**2.379**	0.07	ASV_14_Lachnospiraceae_NA
0.007	**2.354**	0.058	ASV_17_Lachnospiraceae_Fusicatenibacter
**1.526**	–	0.071	ASV_30_Lachnospiraceae_NA
0.053	**4.658**	–	ASV_35_Enterococcaceae_Enterococcus
**2.468**	0.001	–	ASV_41_Lachnospiraceae_Sellimonas
**1.208**	0.008	**2.621**	ASV_44_Peptostreptococcaceae_Romboutsia

ASVs exclusive to T2 bird ileum and abundances	Conv_Abund	Egg_Abund	Env_Abund
ASV_8_Lachnospiraceae_NA	**1.624**	0.378	0.312
ASV_9_Enterococcaceae_Enterococcus	**1.875**	**5.31**	–
ASV_20_Lachnospiraceae_Eisenbergiella	**1.006**	0.248	0.142
ASV_21_Lachnospiraceae_NA	**1.196**	0.386	0.021
ASV_22_Lachnospiraceae_Lachnoclostridium	**1.376**	0.148	0.246
ASV_27_Lachnospiraceae_Blautia	**1.02**	0.269	0.123
ASV_39_Lachnospiraceae_NA	**1.555**	0.419	0.059
ASV_56_Clostridiaceae_1_Clostridium_sensu_stricto_1	0.299	**1.504**	0.204
ASV_70_Clostridiaceae_1_Clostridium_sensu_stricto_1	0.08	0.133	**1.468**

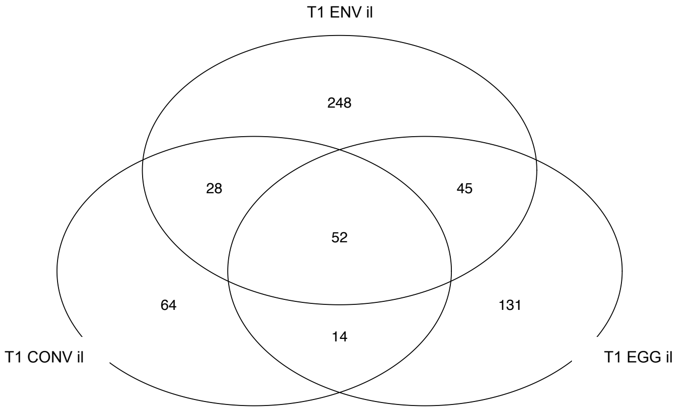

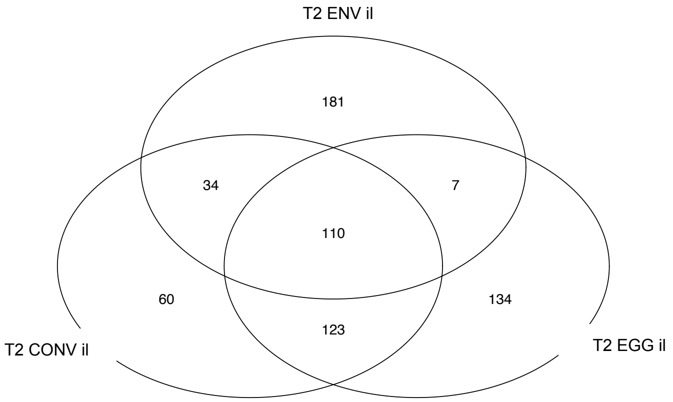

Supplement: Supplementary file 12 — Additional file 12: Figure S7. List of ileum-associated bacterial ASVs shared between bacterial input groups and trials. ASVs are listed with a number followed by the associated family and genus classifications and a number if there are multiple, unique ASVs with the same family and genus classifications. Bolded numbers are > 1.0% relative abundance. Core shared ASVs in the upper table are those present at > 1.0% relative abundance in a least one of the microbial input groups in both trials. ASVs present at > 1.0% relative abundance in a least one of the microbial input groups in only one trial are found in the lower potion of the table. Venn diagrams at the bottom of the figure show the sharing of ASVs between microbial input groups with > 10 reads within a trial. [file 40104_2020_459_MOESM12_ESM.pdf]
