# Supplementary material for: Eggshell and environmental bacteria contribute to the intestinal microbiota of growing chickens
Source: J Anim Sci Biotechnol. 2020 Jun 11;11:60. doi: 10.1186/s40104-020-00459-w (PMC7288515; doi:10.1186/s40104-020-00459-w)

Highly abundant ASVs (>1.0% in at least one input group) shared between T1 and T2 bird jejunum

T1 bird jejunal ASV abundances				T2 bird jejunal ASV abundances		
Conv_Abund	Egg_Abund	Env_Abund	ASV and Taxonomy	Conv_Abund	Egg_Abund	Env_Abund
**13.041**	**47.889**	**17.48**	ASV_1_Enterococcaceae_Enterococcus	**38.35**	**63.211**	**17.832**
**5.227**	0.266	**14.763**	ASV_2_Peptostreptococcaceae_Romboutsia	**1.388**	**1.872**	**18.32**
**36.35**	**3.292**	**3.222**	ASV_3_Lachnospiraceae_NA	**13.404**	0.208	**3.315**
**12.851**	**11.531**	**1.563**	ASV_4_Lachnospiraceae_NA	**5.596**	0.194	**2.644**
0.03	0.017	**15.773**	ASV_5_Clostridiaceae_1_Clostridium_sensu_stricto_1	**7.108**	**11.263**	**4.205**
**3.574**	0.257	**9.415**	ASV_6_Enterobacteriaceae_Escherichia/Shigella	**9.743**	**10.055**	**11.125**
**6.218**	0.321	**1.121**	ASV_7_Lachnospiraceae_NA	**4.441**	0.038	0.055
**2.577**	0.007	0.189	ASV_9_Enterococcaceae_Enterococcus	**1.532**	**7.875**	0.059
0.709	**2.989**	0.289	ASV_10_Enterobacteriaceae_Klebsiella	0.103	0.034	**3.002**
0.574	**2.277**	0.105	ASV_12_Enterococcaceae_Enterococcus	**2.653**	0.293	0.038
0.681	0.026	**1.01**	ASV_23_Peptostreptococcaceae_Romboutsia	0.055	0.097	**1.073**

Highly abundant ASVs (>1.0% in at least one input group) exclusive to either T1 or T2 bird jejunum

			ASVs exclusive to T1 bird jejunum and abundances
**1.333**	**1.28**	0.699	ASV_8_Lachnospiraceae_NA
0.025	–	**4.5**	ASV_13_Erysipelotrichaceae_Turicibacter
0.056	**1.935**	0.294	ASV_14_Lachnospiraceae_NA
**1.545**	0.519	0.061	ASV_16_Ruminococcaceae_Flavonifractor
0.064	**1.955**	0.102	ASV_17_Lachnospiraceae_Fusicatenibacter
**1.74**	0.078	0.263	ASV_30_Lachnospiraceae_NA
0.017	**4.098**	0.003	ASV_35_Enterococcaceae_Enterococcus
**2.135**	0.139	–	ASV_41_Lachnospiraceae_Sellimonas
0	**3.3**	0.001	ASV_42_Enterococcaceae_Enterococcus
0.05	**1.101**	–	ASV_52_Lachnospiraceae_NA
0.048	**1.017**	0.006	ASV_55_Lachnospiraceae_NA

ASVs exclusive to T2 bird jejunum and abundances			
ASV_11_Clostridiaceae_1_Clostridium_sensu_stricto_1	0.25	0.012	**2.536**
ASV_15_Lachnospiraceae_NA	0.103	0.091	**1.821**
ASV_19_Lachnospiraceae_NA	0.102	0.016	**1.882**
ASV_21_Lachnospiraceae_NA	**1.431**	0.004	0.106
ASV_26_Peptostreptococcaceae_Terrisporobacter	0.003	–	**1.134**
ASV_27_Lachnospiraceae_Blautia	**1.249**	0	0.146
ASV_38_Lachnospiraceae_Sellimonas	0.035	0.001	**2.08**
ASV_139_Bacillaceae_Bacillus	0.146	0.184	**1.826**
ASV_141_Burkholderiaceae_Herbaspirillum	0.071	0.013	**1.306**
ASV_164_Burkholderiaceae_Cupriavidus	0.031	0.001	**1.688**

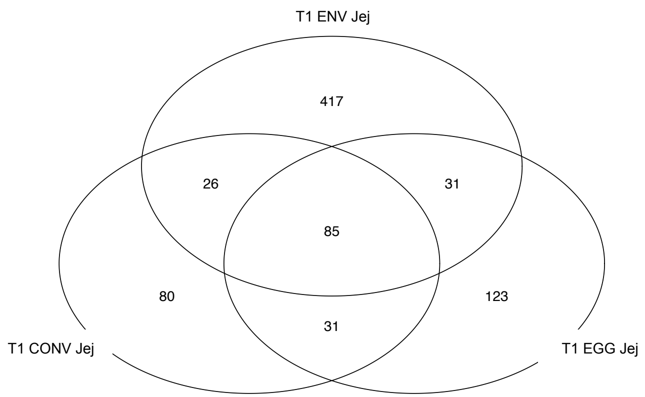

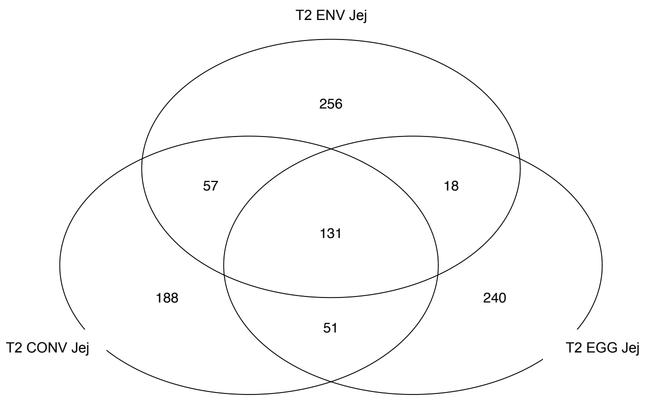

Supplement: Supplementary file 13 — Additional file 13: Figure S8. List of jejunum-associated bacterial ASVs shared between bacterial input groups and trials. ASVs are listed with a number followed by the associated family and genus classifications and a number if there are multiple, unique ASVs with the same family and genus classifications. Bolded numbers are > 1.0% relative abundance. Core shared ASVs in the upper table are those present at > 1.0% relative abundance in a least one of the microbial input groups in both trials. ASVs present at > 1.0% relative abundance in a least one of the microbial input groups in only one trial are found in the lower potion of the table. Venn diagrams at the bottom of the figure show the sharing of ASVs between microbial input groups with > 10 reads within a trial. [file 40104_2020_459_MOESM13_ESM.pdf]
