# Supplementary material for: Eggshell and environmental bacteria contribute to the intestinal microbiota of growing chickens
Source: J Anim Sci Biotechnol. 2020 Jun 11;11:60. doi: 10.1186/s40104-020-00459-w (PMC7288515; doi:10.1186/s40104-020-00459-w)

Highly abundant ASVs (>1.0% in at least one input group) shared between T1 and T2 birds across all samples

T1 bird ASV abundances: Conv_Abund	T1 Egg_Abund	T1 Env_Abund	ASV and Taxonomy	T2 bird ASV abundances: Conv_Abund	T2 Egg_Abund	T2 Env_Abund
**8.929**	**36.467**	**7.759**	ASV_1_Enterococcaceae_Enterococcus	**16.175**	**38.578**	**8.062**
**5.656**	0.607	**16.78**	ASV_2_Peptostreptococcaceae_Romboutsia	**1.154**	**4.757**	**22.177**
**33.583**	**2.781**	**3.156**	ASV_3_Lachnospiraceae_NA	**17.431**	0.555	**2.506**
**12.287**	**14.322**	**2.235**	ASV_4_Lachnospiraceae_NA	**12.351**	**1.056**	0.812
0.015	0.024	**9.046**	ASV_5_Clostridiaceae_1_Clostridium_sensu_stricto_1	**3.426**	**7.934**	**1.965**
**2.351**	0.284	**2.232**	ASV_6_Enterobacteriaceae_Escherichia/Shigella	**9.603**	**9.132**	**2.716**
**7.925**	0.089	0.906	ASV_7_Lachnospiraceae_NA	**5.558**	0.193	0.061
**1.38**	**2.543**	**1.315**	ASV_8_Lachnospiraceae_NA	0.587	**3.566**	**1.427**
**1.443**	0.004	0.048	ASV_9_Enterococcaceae_Enterococcus	0.912	**4.636**	0.068
0.947	**4.21**	**1.106**	ASV_10_Enterobacteriaceae_Klebsiella	0.528	0.031	**4.774**
**1.164**	0.503	**2.322**	ASV_11_Clostridiaceae_1_Clostridium_sensu_stricto_1	0.303	0.105	**5.946**
0.647	**2.103**	0.154	ASV_12_Enterococcaceae_Enterococcus	**3.507**	0.587	0.612
0.107	0.002	**5.544**	ASV_13_Erysipelotrichaceae_Turicibacter	0.002	0.002	**1.686**
0.495	**4.461**	0.318	ASV_14_Lachnospiraceae_NA	0.233	**1.166**	0.212
**1.985**	0.217	0.373	ASV_16_Ruminococcaceae_Flavonifractor	**2.366**	0.066	0.905
0.013	0.803	**1.889**	ASV_19_Lachnospiraceae_NA	0.074	0.184	**2.605**
**1.093**	0.15	**1.064**	ASV_21_Lachnospiraceae_NA	**1.266**	0.091	0.062
0.684	0.005	**1.683**	ASV_26_Peptostreptococcaceae_Terrisporobacter	0.004	0.001	**2.435**
**2.196**	0.018	0.205	ASV_30_Lachnospiraceae_NA	**1.505**	0.027	0.016

Highly abundant ASVs (>1.0% in at least one input group) exclusive to either T1 or T2 birds across all samples

ASVs exclusive to T1 birds and abundances

Conv_Abund	Egg_Abund	Env_Abund	ASV
0.661	**4.575**	0.253	ASV_17_Lachnospiraceae_Fusicatenibacter
0.71	0.055	**1.2**	ASV_23_Peptostreptococcaceae_Romboutsia
0.082	**1.9**	0.425	ASV_25_Ruminococcaceae_Ruminiclostridium_9
0	**NA**	**3.298**	ASV_31_Ruminococcaceae_Subdoligranulum
0	0.001	**1.72**	ASV_34_Ruminococcaceae_Ruminiclostridium_5
0.076	**2.987**	0.001	ASV_35_Enterococcaceae_Enterococcus
**2.423**	0.046	0.001	ASV_41_Lachnospiraceae_Sellimonas
0.03	**2.547**	0	ASV_42_Enterococcaceae_Enterococcus
0.456	0.033	**1.36**	ASV_44_Peptostreptococcaceae_Romboutsia
0.199	**1.334**	0.001	ASV_52_Lachnospiraceae_NA
0.285	**1.335**	0.003	ASV_55_Lachnospiraceae_NA

ASVs exclusive to T2 birds and abundances

ASV	Conv_Abund	Egg_Abund	Env_Abund
ASV_15_Lachnospiraceae_NA	0.283	**1.598**	**3.559**
ASV_20_Lachnospiraceae_Eisenbergiella	0.495	**1.544**	0.647
ASV_22_Lachnospiraceae_Lachnoclostridium	**1.582**	0.058	0.544
ASV_24_Bacillaceae_NA	0.092	**1.007**	0.42
ASV_27_Lachnospiraceae_Blautia	**1.202**	0.064	0.473
ASV_28_Lachnospiraceae_NA	0.421	**1.472**	0.677
ASV_29_Lachnospiraceae_NA	0.085	0.377	**3.052**
ASV_36_Lachnospiraceae_NA	0.148	**1.039**	0.602
ASV_38_Lachnospiraceae_Sellimonas	0.04	0.028	**2.844**
ASV_39_Lachnospiraceae_NA	**1.895**	0.118	0.025
ASV_40_Lachnospiraceae_Sellimonas	0.027	0.006	**1.216**
ASV_45_Peptostreptococcaceae_Romboutsia	0.02	0.219	**1.128**
ASV_70_Clostridiaceae_1_Clostridium_sensu_stricto_1	0.038	0.043	**1.136**

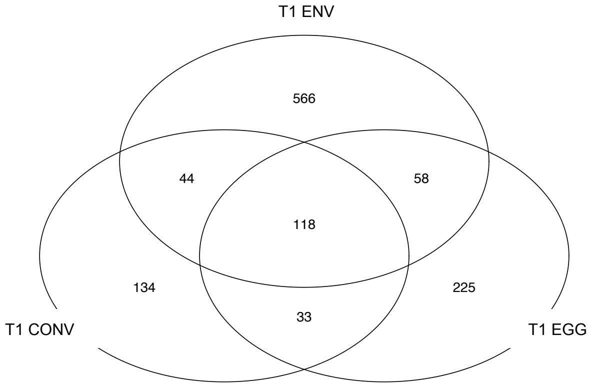

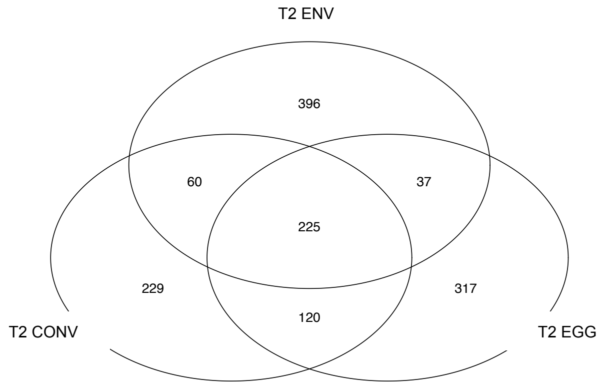

Supplement: Supplementary file 14 — Additional file 14: Figure S9. List of all bacterial ASVs shared between bacterial input groups and trials. ASVs are listed with a number followed by the associated family and genus classifications and a number if there are multiple, unique ASVs with the same family and genus classifications. Bolded numbers are > 1.0% relative abundance. Core shared ASVs in the upper table are the core ASVs shared between both trials present at > 1.0% relative abundance in a least one of the microbial input groups in both trials and present at > 10 reads in all the microbial input groups in both trials. ASVs present at > 1.0% relative abundance in a least one of the microbial input groups and present at > 10 reads in all the microbial input groups within only one trial are found in the lower potion of the table. Venn diagrams at the bottom of the figure show the sharing of ASVs between microbial input groups with > 10 reads within a trial. [file 40104_2020_459_MOESM14_ESM.pdf]
